# Human players manage to extort more than the mutual cooperation payoff in repeated social dilemmas

**DOI:** 10.1038/s41598-021-96061-9

**Published:** 2021-08-19

**Authors:** Chiara D’Arcangelo, Luciano Andreozzi, Marco Faillo

**Affiliations:** 1 Dipartimento di Economia, Università degli Studi G. D’Annunzio Chieti-Pescara, 65127 Pescara, Italy; 2 Dipartimento di Economia e Management, Università di Trento, 38122 Trento, Italy

**Keywords:** Human behaviour, Social evolution

## Abstract

Social dilemmas are mixed-motive games. Although the players have a common interest in maintaining cooperation, each may try to obtain a larger payoff by cooperating less than the other. This phenomenon received increased attention after Press and Dyson discovered a class of strategies for the repeated prisoner’s dilemma (extortionate strategies) that secure for themselves a payoff that is never smaller, but can be larger, than the opponent’s payoff. We conducted an experiment to test whether humans adopt extortionate strategies when playing a social dilemma. Our results reveal that human subjects do try to extort a larger payoff from their opponents. However, they are only successful when extortionate strategies are part of a Nash equilibrium. In settings where extortionate strategies do not appear in any Nash equilibrium, attempts at extortion only result in a breakdown of cooperation. Our subjects recognized the different incentives implied by the two settings, and they were ready to “extort” the opponent when allowed to do so. This suggests that deviations from mutually cooperative equilibria, which are usually attributed to players’ impatience, coordination problems, or lack of information, can instead be driven by subjects trying to reach more favorable outcomes.

## Introduction

In a prisoner’s dilemma (PD), two players must decide whether to cooperate or to defect. Although their payoff is larger if they both cooperate, each of them has an incentive to defect. This game is interesting because if it is played only once, mutual defection is the only rational course of action, but, if it is played repeatedly and with a sufficiently long time-horizon, cooperation becomes a Nash equilibrium (NE). For this reason, over the years, the repeated version of the PD has become a fundamental element in studying the emergence of cooperation, both in biology^1–10^ and in the social sciences^11–15^.

In a seminal paper, Press and Dyson^16^ attracted scholars’ attention to a somewhat neglected issue. They noticed that when a social dilemma is played repeatedly, there is always some conflict of interest because each player is tempted to cooperate less than the other to reap a larger share of the gains from cooperation. This result was not novel. One of the best-established results in game theory, the so-called *folk-theorem*, shows that when a game like the PD is repeated with a sufficiently long time-horizon, a host of new equilibria emerge^17^. In most of them, one player obtains a larger payoff than the other. The novelty of Press and Dyson’s result is that the repeated PD contains a class of strategies that they termed *extortionate strategies* (ExS), which secure for themselves a payoff that is never smaller (but may be larger) than the opponent’s, regardless of the strategy that the latter may employ. In this sense, ExS are *unbeatable*^18^. What makes this result particularly intriguing is that ExS are simple memory-one strategies: they base their current decision only on the outcome of the previous round.

The literature that followed pointed out several weak points of this result^19^. First, since the PD is a symmetric game, asymmetric outcomes are hard to justify. Press and Dyson had already noticed that when two ExS are matched against each other, no extortion takes place because they both get the mutual defection payoff. This means that ExS can only succeed when they meet strategies that are not ExS, and hence they can never become dominant within a single population. This has led some authors to question whether ExS can play any significant role in the emergence of cooperation^18,20–27^.

Second, in a standard PD, ExS are difficult to compute, which partly explains why they have long-escaped scholars’ attention. This issue is particularly relevant for experimental contexts in which human behavior is observed, because it is unlikely that bounded rational players discover them by trial and error. A few experiments have investigated how humans react when facing ExS, by observing how they play against computers programmed to play such strategies^18,28^. Much less is known about whether human beings are able to discover them. To the best of our knowledge, the only study that has investigated whether human subjects adopt ExS in experimental settings is Becks and Milinski^29^. Their study reveals that ExS are observed only when an extra reward is given to the player who is able to gain more than the opponent. The authors concluded that ExS can only prevail when higher competitiveness is rewarded with extra gain.

Finally, a question left open by Press and Dyson is whether ExS can be part of a NE when players are constrained to play memory-one strategies. This question is important because, if the answer is negative, ExS are bound to play a minor role in evolutionary models, even in asymmetric, multi-population settings^22,30,31^ and may be more difficult to observe in experiments^32^.

These considerations suggest that ExS are more likely to play a role, both in theoretical models and in laboratory experiments, in settings that differ from the standard PD, at least in these three respects. First, we should look for asymmetric games where one player is in a better position to extort a larger payoff. Second, these games should contain ExS that are intuitive and relatively easy to discover. Finally, these ExS must be part of a NE.

**Figure 1:**
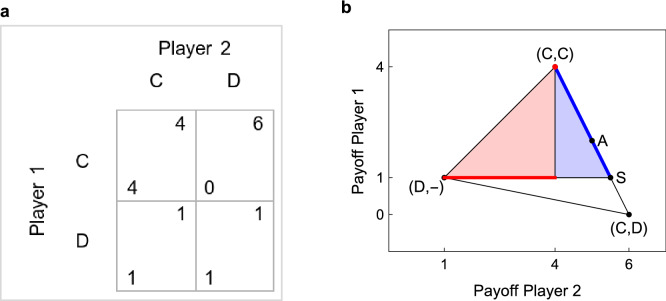
The STG we used for our experiment (**a**), and the set of feasible payoff profiles (**b**). If the game is played repeatedly and the players are sufficiently patient, every point in the colored areas can be sustained in a (subgame perfect) NE. Points in the blue area correspond to extortionate equilibria; that is, equilibria in which Player 2 obtains more than the mutual cooperation payoff. If Player 1 is constrained to memory-one strategies, extortionate equilibria exist only if she can observe the mixed strategy chosen by Player 2. If Player 1 only observes the outcome of the previous round, the only payoff profiles that can be sustained in equilibrium are those in dark red. In none of them Player 2 gets more than the mutual cooperation payoff, so no extortion can occur.

To address these issues, we consider the Simultaneous-move Trust Game^33,34^ (STG) shown in Fig. 1a. Differently from the sequential version of the Trust Game, in this game Player 2 makes his decision without observing Player 1’s choice. As in the PD, the players of our game choose between Cooperation (C) and Defection (D), and the payoff for mutual cooperation is higher than the payoff for mutual defection. The game has a single, pure strategy NE in which both players choose D, and D is a (weakly) dominant strategy for Player 2. Unlike the PD, however, D is not a dominant strategy for Player 1, as C is the best response to C.

Figure 1b represents the STG’s set of feasible payoff profiles. Profiles in the colored areas are those in which both players obtain more than the mutual defection payoff. Profiles in the blue area are those in which Player 2 obtains more than the mutual cooperation payoff. In this case, we say that Player 2 enforces a *payoff premium*. We label a strategy as extortionate if it is able to enforce a payoff premium when it is matched against its best reply. In contrast to the PD, in the STG only Player 2 can have ExS, as Player 1 can never get more than the mutual cooperation payoff. A second crucial difference is that the STG includes a very simple and intuitive class of ExS. These strategies prescribe to play C with a fixed probability *q*, independently from the previous history of the play. For this reason they are referred to as *unconditional* strategies. For an unconditional strategy to be ExS, the probability *q* must be sufficiently large to make C the best reply for Player 1. In the game in Fig. 1, *q* must be larger than $\frac{1}{4}$ (see “Methods”). For example, under the assumption that Player 1 plays the best reply to whatever strategy the opponent plays, Player 2 can reach the payoff profile A in Fig. 1b by equally randomizing between C and D at each round, regardless of the other player’s previous choices. It is easy to show, however, that if Player 1 can base her choice in the current round only on the outcome of the previous one, there is no NE in which she always cooperates, while Player 2 cooperates in half of the rounds, so that the resulting payoff profile would be A. To see this, consider that the best reply of Player 1 to Player 2’s strategy is to play C in response to C *and* in response to D. But if this is the case, then the optimal choice for Player 2 is to play D always, rather than in half of the rounds. The reason is that Player 1 (who only takes into account the previous outcome) cannot punish deviations from a mixed strategy, which proves that the initial strategy profile was not a NE. This result holds in general: as long as Player 1 can base her decision only on the previous round’s outcome, no NE exists in which Player 2 gets more than the mutual cooperation payoff (see “Methods”).

Our discussion thus far illustrates a weakness that all ExS share. To get more than the mutual cooperation payoff, Player 2 must play C with a probability $q^{*}$ smaller than one. However, for this to be part of a NE, Player 1 should be able to punish Player 2 (by playing D) for choosing C with a probability smaller than $q^{*}$, something that cannot be done if she can base her decision only on the previous outcome of the game.

This suggests that a version of the Press and Dyson result may be restored if players can observe not only the outcome of the previous round but also the opponent’s mixed strategy. In the literature, these are known as repeated games with observable mixtures^17^. It is not difficult to see that if mixtures are observable, then extortion can take place in equilibrium, even when Player 1 is constrained to memory-one strategies^35^. In this case, in our game there is a family of Nash equilibria in which, at every round, Player 2 chooses C with a probability ${q}^{*}$, with $1 \ge {q}^{*}\ge \frac{1}{4}$, and Player 1 chooses C only if, in the previous round, Player 2 chose C with a probability $q \ge {q}^{*}$ (see “Methods”). These equilibria are intuitively appealing, because they represent the situation in which Player 2 builds a reputation for being sufficiently cooperative (${q}^{*} \ge \frac{1}{4}$) while Player 1 punishes Player 2 if he fails to live-up to his reputation (that is, if she observes $q<{q}^{*}$).

Our theoretical result has two empirically verifiable consequences. First, if Player 1 can base her choice only on the outcome of the previous round, then no extortion can be successful. Second, if Player 1 can base her choice on the mixed strategy that was played by Player 2 in the previous round, then extortionate equilibria are possible, and Player 2 can get more than the mutual cooperation payoff, enforcing a payoff premium.

An obvious difficulty in testing these predictions is determining how to force subjects to use memory-one strategies. When playing repeatedly against the same opponent, a human subject will typically make choices based on the game’s entire history, not just on the previous round. This implies that in our experiment we could not use a standard repeated game played between partners (i.e. having the same two players interacting in every period). We circumvented this problem with the following experimental design. At the beginning of the experiment, subjects were equally divided to play either as Player 1 or Player 2, and they maintained the same role for the experiment’s entire duration. The experiment lasted for a variable number of rounds, not known to the subjects. At each round, subjects were randomly matched to play the STG in Fig. 1a (this implies that we are in a between-stranger setting, where each player interacts with a different opponent at every round). Before making a choice, each Player 1 was given information about the previous behavior of Player 2, with whom she was matched within the current round (see “Methods”).

Depending on the type of information that we disclosed, two classes of treatments were obtained. In the Outcome treatments, Player 1 observed the action (that is, either C or D) that Player 2 chose in the previous round. In the Mixture treatments, Player 1 observed the frequency with which Player 2 played C in all the previous rounds.

The link between our experimental design and the original Press and Dyson result is provided by our final theoretical result. We prove that the set of Nash equilibria for the Mixture and Outcome treatments (our stranger setting) is a subset of the set of Nash equilibria for a standard repeated STG (that is, played between partners) in which Player 1 bases her choice either on the previous round outcome (in the Outcome treatments) or on the mixed strategy chosen by Player 2 (in the Mixture treatments).

The intuition behind this result is straightforward. From the point of view of Player 2, it makes no difference whether he faces a single memory-one Player 1 who only remembers the strategy Player 2 choose in the previous round, or a succession of different Players 1, each of whom can observe the strategy Player 2 used with the previous Player 1. From the point of view of Player 1, C is the best reply in the current round whenever she expects Player 2 to play C with a sufficiently large probability. It follows that if to play C is optimal for a patient Player 1 who interacts repeatedly with the same Player 2, it will also be optimal for a succession of Players 1, each of whom only plays once. In both cases, Player 1 will play a myopic best reply to the (mixed) strategy she expects Player 2 to play in the current round.

Our theoretical result implies that Extortionate equilibria only exist in the Mixture treatments, and this gives us the following predictions:

**Prediction1** Player 2 can enforce a payoff premium in the Mixture, but not in the Outcome treatments.

**Prediction2** ExS are more frequent in the Mixture than in the Outcome treatments.

The results from our experiment are in surprisingly good agreement with these predictions.

## Results

### Strategies elicitation

A common problem with experiments involving repeated games is how to infer the repeated game strategies employed by the subjects from the actions (either C or D) they choose during the game. This problem is particularly acute in an experiment like ours, in which subjects were expected to use mixed strategies. To address this issue, we elicited subjects’ strategies by using two different approaches. In the Direct Response Method (DRM), subjects chose an action at each round of the game. In the Strategy Method (SM), subjects submitted their strategies in the form of simple computer programs^32,36,37^. The actual play of the repeated game was then carried out by the computer, and each subject received the payoff obtained by the program he chose (see Table 1 and “Methods” for further details).

**Table 1:** Overview of the experiment. We elicited subjects’ strategies using two different methods.

Strategy elicitation	Player 2	Player 1	
		Mixture treatment	Outcome treatment
DRM (direct response method)	In every period, he decides whether to play C or D	In every period $t>1$, she observes the *frequency* with which her current opponent played C in all the previous periods, and decides whether to play C or D	In every period $t>1$, she observes the *action* played by her current opponent in the previous period, and decides whether to play C or D
SM (strategy method)	At the beginning of the game, he chooses a mixed strategy, (i.e. a probability with which the computer will play C at every round) in the set $\{0, 0.25, 0.5, 0.75, 1\}$	At the beginning of the game, she instructs the computer to play C or D for each possible mixed strategy that Player 2 could choose	At the beginning of the game, she instructs the computer to play C or D, for each possible realization (C or D) of the mixed strategy chosen by Player 2

In the Direct Response Method, subjects chose in every period whether to play C or D. In the Strategy Method, subjects submitted their strategies only once, in the form of simple computer programs. For each Method, we run two treatments. In the Mixture treatments, subjects playing as Players 1 could base their decision on the *frequency* with which the opponent played C in the previous periods. In the Outcome treatments, they could base their decision only on the *action* taken by their opponent in the previous period. Note that subjects playing as Players 2 had the same strategy set in both treatments.

### Extortionate outcomes

**Figure 2:**
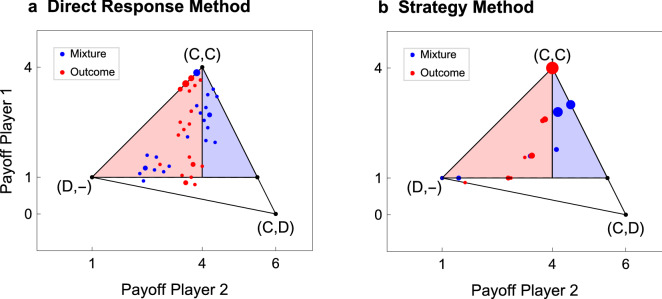
Players’ payoff profiles. We elicited subjects’ strategies in two different ways. In the DRM, decisions were made at each round of the repeated game (**a**). In the SM, subjects submitted their strategies in the form of simple computer programs (**b**). Note that, since in the SM players 2 could choose among a predetermined set of strategies (see Table 1) data points are less dispersed in this case. The coordinates of each dot are the average payoff of a single Player 2 (x-axes) and the average payoff that Players 1 obtained when they interacted with him (y-axes). Dots are red when Player 1 can observe only the action that was chosen by Player 2 in the previous round (i.e., Outcome treatment, $n=27$); they are blue when Player 1 observed the frequency with which Player 2 chose the different actions in all the previous rounds (i.e., mixture treatment, $n=29$). Dots’ size is proportional to the number of occurrences of that data point. Our theory predicts that all red dots should be in the red area, while blue dots may appear in the blue area. The results from our experiment are broadly in agreement with these predictions.

Figure 2 shows the main result of our experiment. It represents the payoff profiles that the subjects obtained in our treatments. The coordinates of each dot represent the average payoff of a single Player 2 (x-axes), and the average payoff that Players 1 obtained when they interacted with him (y-axes). In the DRM, these are just the average payoffs that players obtained during the first 20 rounds of the game. In the SM, the actual choice (either C or D) of Player 2 was determined by the computer, according to his chosen program. In this case, we computed the expected payoff of the programs that were submitted by each Player 2, given the strategies submitted by the Players 1 with whom he had interacted (see “Methods” for further details).

Figure 2 shows that all Players 2 who managed to enforce a payoff premium (that is, who were able to get, on average, more than the mutual cooperation payoff) belonged to the Mixture treatment. Specifically, in the Mixture treatment, 10 subjects (34%) in the DRM and 17 subjects (59%) in the SM were able to extort a payoff larger than 4, the mutual cooperation payoff, while none of them did so in the Outcome treatment (Fisher’s exact test for the difference between treatments: $p = 0.001$ (DRM); $p < 0.001$ (SM)). This resulted in a larger payoff for Players 2 in the Mixture treatment. In the DRM, the median payoffs of Player 2 in the Mixture and in the Outcome treatments are 3.84 and 3.65, respectively (Pearson $chi2 = 6.2237$, $p = 0.013$). This tendency is even more pronounced in the SM, as the median payoffs in the Mixture and the Outcome treatments are 4.14 and 3.8, respectively (Pearson $chi2 = 20.0386$, $p < 0.001$). Thus, not only Players 2 were able to enforce a payoff premium only in the Mixture treatments, but, when we consider the SM, their median payoff (in the Mixture treatment) is above the mutual cooperation payoff.

#### Finding 1

In accordance with our Prediction 1, Players 2 are more likely to enforce a payoff premium in the Mixture, rather than in the Outcome treatments.

### Players 2’s strategies

**Figure 3:**
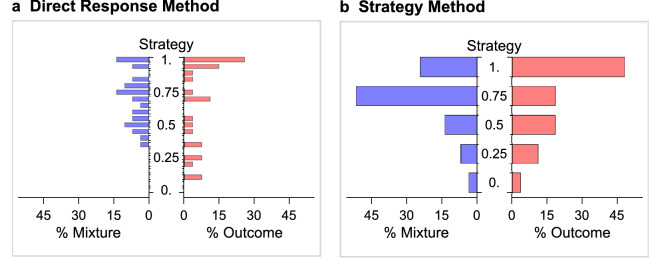
Distribution of Players’ 2 strategies. The histogram bars represent the frequency with which each strategy is played within the population of Players 2. In the DRM, a strategy is the fraction of periods in which Player 2 plays C, while in the SM a strategy is the one submitted by Player 2. In the Outcome treatments ($n=27$), the most frequent strategy is the fully cooperative one, in which C was chosen throughout the repeated game. In the Mixture treatments ($n=29$), 0.75 is a modal choice in both the DRM and the SM. This is in accordance with our Prediction 2.

Figure 3 shows our second result. We classified Players 2 according to our elicited strategy. In the DRM, we considered the number of times that a subject had played C during the first 20 rounds of the game, which gave us 21 possible strategies (cooperation rates over time and by treatments are shown in Supplementary Information 1). For example, we say that a subject used the strategy 0.6 if he played C 12 times out of 20 rounds, while we say that he used the fully cooperative strategy 1 if he played C in all the 20 rounds.

In the SM, we just considered the strategy submitted by Player 2, that is, the probability with which a subject had instructed the computer to play C. Since we allowed Player 2 to choose among five probabilities (see Table 1), we have five possible strategies. Figure 3 represents percentage of play of each strategy in the various treatments (Epps–Singleton Two-Sample test: $W2 = 13.926$, $p = 0.00753$ (a); $W2 = 8.180$, $p=0.08520$ (b)).

Figure 3 conveys two main messages. First, in the Outcome treatments, the modal choice is outright cooperation: 26% of Players 2 in the DRM and 48% in the SM chose to play C in every period of the game. By contrast, in the Mixture treatments, these numbers are down to 14% in the DRM and 24% in the SM (although only in the latter case the difference is significant: 1-sided proportion test for the difference between treatments, $p = 0.0305$ (SM)).

Second, more subjects chose an ExS in the Mixture than in the Outcome treatments. In the Mixture treatments, 86% of Players 2 in the DRM and 72% in the SM chose to play C with a frequency of at least 25% (but smaller than one). Recall that, in our game, a strategy is extortionate if it prescribes to cooperate with a probability larger than . Remarkably, only one subject in the Mixture treatment chose to cooperate with a smaller probability. In the Outcome treatments, hich we also used t ExS were chosen by 63% of Players 2 in the DRM and by 48% in the SM (1-sided proportion test for the difference between treatments: $p = 0.0224$ (DRM), $p = 0.0316$ (SM)).

Among the ExS, in the Mixture treatments the most frequent is 0.75, that is, the one that played C 75% of the times (this strategy was chosen by 14% of Players 2 in the DRM and by 52% in the SM). On the contrary, in the Outcome treatments, the most chosen ExS was 0.95 (that played C 95% of the times) in the DRM (chosen by 15% of Players 2), while in the SM, both 0.5 and 0.75 were chosen by 19% of Players 2. This finding is interesting because it shows that some Players 2 were trying to enforce a payoff premium by using an extortionate strategy, even in a context where they could not succeed because mixed strategies were not observable. Further details on Players 2’s strategies are provided in ‘Supplementary Information”.

#### Finding 2

In accordance with our Prediction 2, full cooperation was the (only) modal choice in the Outcome treatments, while in the Mixture treatment the modal choice was (also) an extortionate strategy.

### Players 1’s strategies

We classified Players 1 according to the strategy that they chose in reaction to the information that they had received. For each treatment, we assumed that subjects used the strategies that appear in our theoretical model. (The details of the classification are in “Supplementary Information”).

**Figure 4:**
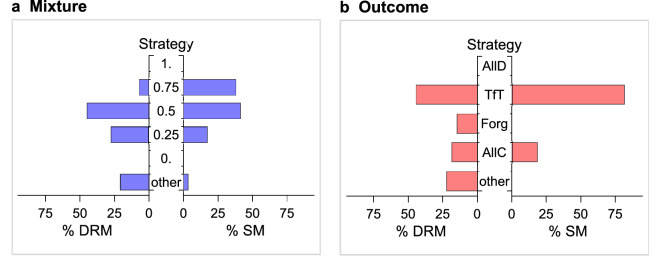
Distribution of Players’ 1 strategies. Players 1 were classified according to their choices in reaction to the information they had received in each treatment. In the Mixture treatments ($n=29$), the most frequent strategy was 0.5, that is, subjects who played C only if the observed frequency of cooperation was at least 50%. In the Outcome treatments ($n=27$), the most frequent strategy was TfT. This is in accordance with Player 1 choosing (one of his) equilibrium strategies.

In the Mixture treatments, this implies that Players 1 used a step strategy, which cooperates only if, in the previous round, Player 2 cooperated with a probability larger than a threshold ${q}^{*}$, and defect otherwise. In the SM, this gives us five possible step strategies for Player 1 (see “Methods”), which we also used to classify subjects in the DRM. Figure 4a shows the percentage of play of each of the five possible step strategies in the Mixture treatments. Note that in the SM, virtually all subjects chose a step strategy. In the DRM, a reasonably large number of subjects (80%) could also be classified in this way. The two distributions, when considering only the subjects that we could classify, are remarkably similar and in agreement with our theoretical predictions. In both cases, no subject chose to play C in response to an observed probability smaller than 0.25, and, more importantly, no subject chose to play C only in response to full cooperation. They were all willing to tolerate some extortion. The modal choice in both cases was to play C in response to a cooperation probability of at least 0.5, although subjects were more willing to tolerate extortion in the DRM than in the SM. Around 20% of the subjects (28% in the DRM, and 17% the in SM) were willing to accept the least favorable probability of cooperation (0.25).

For the Outcome treatments, we classified subjects as: AllD and AllC (they played D or C, regardless of the observed behavior), TfT (they played C if they had observed C, while if they had observed D, they played C with a probability smaller than $\frac{1}{3}$), and Forgiver (“Forg”, they played C if they observed C, but if they had observed D, they played C with a probability larger than $\frac{1}{3}$). The distinction between TfT and Forgivers was motivated by the fact that a cooperative equilibrium can only be sustained if a defection of Player 2 is punished by Player 1 by cooperating with a probability smaller than $\frac{1}{3}$ (see “Methods”). Figure 4b shows the classification results for the Outcome treatments. While all subjects in the SM fall into one of these categories, the classification failed for 22% of the subjects in the DRM. However, among the subjects who could be classified, the distribution is remarkably similar and in broad accordance with our theoretical predictions. In both treatments, TfT is by far the most chosen strategy. Remarkably, no subject could be classified as AllD.

#### Finding 3

In the Mixture treatments, all Players 1 were willing to tolerate some extortion. In the Outcome treatments, the modal strategy was TfT.

### Cooperation rates and payoffs

**Figure 5:**
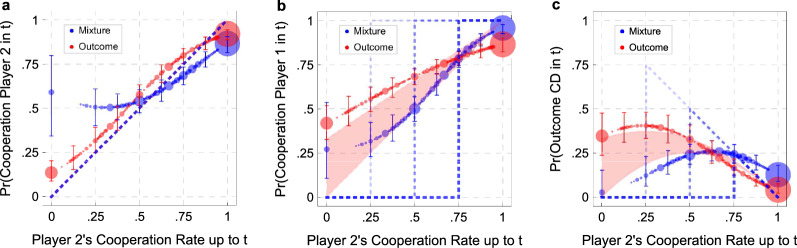
Cooperation rates—direct response method (DRM). On the x-axis we put the frequency with which a given Player 2 chose C in the first $t-1$ rounds. On the y-axis we put: the probability with which he chose C at round *t* (**a**); the probability with which the Player 1 he interacted with at round *t* chose C (**b**), and the probability of observing the outcome CD at round *t* (**c**). Dots’ size is proportional to the number of occurrences of that data point. The red areas and the blue dotted lines correspond to our theoretical predictions for the Outcome and Mixture treatments, respectively. The results are in broad agreement with our theory, with an important difference: in the Outcome treatment, Players 2 that played C with a probability smaller than 0.5 induced a larger than expected rate of cooperation. Detailed estimation procedures, results, and raw data are reported in Supplementary Information 1. n(Mixture) = 551; n(Outcome) = 513.

We now explore in more detail how the strategy chosen by each Player 2 influenced the behavior of the Players 1 with whom he interacted. We shall start with the Direct Response Method. Figure 5 shows the results of a series of logistic regressions in which the dependent variables were Player 2’s choice at round *t* (Fig. 5a), Player 1’ choice at round *t* (Fig. 5b), and the probability of observing the outcome CD at round *t* (Fig. 5c). The independent variable is always the rate of cooperation of Player 2 up to round $t-1$.

The red areas and the blue dotted lines represent our theoretical predictions for the Outcome and the Mixture treatments, respectively. We derived these predictions under the hypothesis that Players 2 used the strategies that appear in our theoretical model. These strategies are unconditional, since at every round they play C with the same probability. This implies that in both treatments, the probability that Player 2 plays C at round *t* should be equal to his cooperation rate up to $t-1$; hence, both red and blue points in Fig. 5a should be located along the dotted line. The data are remarkably consistent with this hypothesis, especially if attention is restricted to probabilities larger than .5, where most of the points are located. (The data for the Mixture treatment diverge from the predicted ones for low values of the cooperation probability, but this can be a consequence of the small number of observations for these probabilities—see Supplementary Information 1).

Players 1’s equilibrium behavior depends on the treatment considered. In the Outcome treatments, in all the cooperative equilibria Player 1 chooses C with probability 1 after observing C, and punishes defection by cooperating with a probability $p_D\le \frac{1}{3}$ after observing D (see “Methods”). Thus, if a Player 2 chooses C with probability *q*, the average cooperation rate of Players 1 would be $q + (1-q)p_D$, where $p_D$ can be any number between zero and $\frac{1}{3}$. The average probability to observe the outcome CD is then $(1-q)(q + (1-q)p_D)$. From this, it follows that red dots should be found in the red areas of Fig. 5b,c.

In the Mixture treatments, if Player 1 uses a step strategy, there should be a threshold ${q}^{*} \ge .25$ such that Player 1 chooses C if the observed probability with which Player 2 cooperated is larger than ${q}^{*}$, and D otherwise. In Fig. 5b,c, the blue dotted lines represent our theoretical predictions, assuming that Players 1 use a step function with a threshold ${q}^{*}=0.25$ (lighter line), ${q}^{*}=0.5$, or ${q}^{*}=0.75$ (darker line). For values of *q* that are larger than the threshold, but smaller than 1, we expect the probability of observing Player 1 to play C to be larger in the Mixture than in the Outcome treatments, while the opposite holds for values of *q* smaller than the threshold. To see this, consider as an example the case in which Player 1 uses a step strategy with threshold ${q}^{*}=0.75$ in the Mixture treatment, and a strategy with $p_C=1$ and $p_D=0$ in the Outcome treatment. Thus, while in the Mixture treatment we would expect full cooperation whenever $q\ge 0.75$, and full defection whenever $q<0.75$, in the Outcome treatment we expect the cooperation of Player 1 to be linearly increasing in the observed cooperation of Player 2 (and equal to $q p_C +(1-q)p_D=q$). This implies that we would expect higher levels of cooperation in the Mixture than in the Outcome when $q>0.75$, and the opposite when $q<0.75$. Note that this holds for any value of $p_D<1$.

Since all step strategies with $0.25\le {q}^{*}\le 1$ are able to form a Nash equilibrium in the Mixture treatment, when looking at the aggregate behavior as showed in panels b and c, we expect the blue dots to be above the red dots for *q* sufficiently close to one, and below otherwise. The data clearly support this conclusion. Specifically, after observing a value of $q>0.75$, the probability that Player 1 plays C is higher in the Mixture than in the Outcome treatment (t test: $t= 20.07$, $p < 0.01$), while the opposite holds after observing a value of $q<0.75$ (t test: $t= 6.24$, $p < 0.01$).

**Figure 6:**
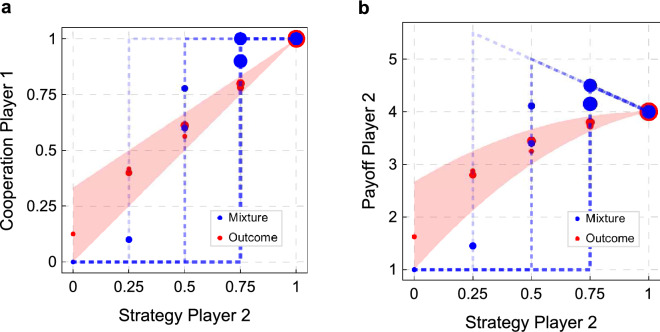
Cooperation rates and payoffs—strategy method (SM). On the x-axis we put the strategy chosen by each Player 2. On the y-axis we put: the frequency with which Player 2 induced the Players 1 he interacted with to play C (**a**), and the payoff Player 2 obtained (**b**). Dots’ size is proportional to the number of occurrences of that data point. Our theoretical model predicts blue points to be close to the dotted lines and red dots to be in the red areas. They are in remarkably good agreement with the observed behavior. n(Mixture) = 29; n(Outcome) = 27.

In the SM there is no need to infer strategies from players’ choices, as strategies are directly submitted by the subjects. Figure 6 shows the cooperation rate of Player 1 (panel a) and the payoff of Player 2 (panel b) as a function of the strategy chosen by Player 2. The theoretical predictions are the same as in the DRM. For this reason, the blue dotted lines and the red areas in Fig. 6 are calculated with the same method we used for Fig. 5. Consider again as an example the case in which Player 1 uses a step strategy with threshold ${q}^{*}=0.75$ in the Mixture treatment, and a strategy with $p_C=1$ and $p_D=0$ in the Outcome treatment. In the previous paragraph we noted that in this case when Player 2 cooperates with a probability *q* larger than 0.75, but smaller than 1, we would expect the probability of observing Player 1 to play C to be larger in the Mixture than in the Outcome treatments, while the opposite holds for values of *q* smaller than 0.75. This implies that for $q>0.75$ ($q<0.75$) the payoff of Player 2 would be larger (smaller) in the Mixture than in the Outcome treatment. Since all step strategies with $0.25\le {q}^{*} \le 1$ can sustain a NE in which Player 1 cooperates, the model cannot pin down the threshold at which we observe this reversal. Figure 6 reveals that there is such a reversal and the threshold is located at about $\bar{q}= 0.5$ (Wilcoxon rank-sum Mann–Whitney test $p < 0.01$)). The same holds for the payoff of Player 2 (Wilcoxon rank-sum Mann-Whitney test $p < 0.01$).

We summarize these results in the following:

#### Finding 4

In accordance with our theoretical predictions, Players 2 using extortionate strategies that cooperate often enough (more than $75\%$ in the DRM, and more than $50\%$ in the SM) were able to induce more cooperation from Players 1 in the Mixture than in the Outcome treatment.

## Discussion

As we said in “Introduction”, when a social dilemma is repeated over time, there is always some conflict of interest between the players. The standard repeated PD downplays the importance of this conflict because it is a symmetric game and contains a symmetric, cooperative equilibrium, which is an obvious focal point for the players^38^. In asymmetric games, such as the STG we considered in this study, it is easier to see that mutual cooperation is not the only equilibrium candidate. An obvious reason is that in such an equilibrium, Player 1 achieves the highest payoff that she can aspire to, while Player 2 may aim at a larger payoff.

These considerations suggest that our results should be compared with the large experimental evidence on bargaining games, in which observed behavior is also known to be influenced by non-selfish motives such as fairness and inequity aversion. In this view, the repeated STG with observable mixtures should be seen as an ultimatum game in which, by choosing a probability of cooperation, Player 2 makes a offer to Player 1 on how to divide the benefits of cooperation. Offers vary in their degree of fairness, depending on how much cooperation Player 2 returns to Player 1. Player 1 can reject any proposal by defecting, although rejecting an offer is costly, as long as Player 2 cooperates with a sufficiently large probability.

Our results are partly consistent with the large evidence on the ultimatum game, with some important differences that suggest that in the repeated STG, fairness considerations play a considerably smaller role than in the ultimatum game. When mixtures were observable, all subjects in our sample were willing to accept a measure of exploitation. Even more striking is the fact that roughly 20% of the subjects would accept even the least favorable offer, in which Player 2 gets all the cooperation benefit. By contrast, in the ultimatum game, it is common to find a non-negligible fraction of subjects who reject any offer below the equal split, while the percentage of subjects who are willing to accept the most unfavorable offers is rarely larger than 10%. Finally, in both Mixture treatments, the modal choice for Players 2 was to extort a larger payoff by cooperating with a probability of 0.75; while in the ultimatum game, it is common to find the equal division as the modal choice among the proposers^39,40^.

We conclude that human subjects do try to extort a larger payoff when in the position to do so. In fact, some subjects try to obtain this result even in situations where they are bound to fail because there are no equilibria in which extortion can take place. This result is important in that it improves our understanding of how repetition and players’ reputation provide a solution to social dilemmas. Failures in securing a cooperative equilibrium are usually attributed to players’ impatience, coordination problems, or lack of information, as is the case in games with imperfect monitoring. Our results reveal that sometimes subjects try to secure for themselves a larger share of the benefits of cooperation and the conflict that ensues determines a breakdown of cooperation. This topic has received little attention thus far, but it surely deserves more attention in the future.

## Methods

### Preliminaries

We consider a general version of the STG in Fig. 1**a**. The action set for both players is denoted by $M=\{C,D\}$. Following the standard notation for the PD, we denote with *T* the temptation payoff, with *S* the sucker’s payoff, with *R* the reward for mutual cooperation, and with *P* the punishment for mutual defection. The game is played repeatedly, with a continuation probability equal to $\delta \in (0,1)$. The set of possible outcomes is $W=\{CC,CD,DC,DD\}$ where, for example, CD is the outcome in which Player 1 cooperates and Player 2 defects, and *w* is a generic element of *W*. $m=(p,q)$ is a generic mixed strategy profile for the stage game, where *p* and *q* are the probabilities with which Player 1 and Player 2 play C respectively. For the repeated game, we denote with $(w_t,m_t)$ the *mixture outcome* of the game, where $m_t$ is the mixed strategy profile chosen at round *t* and $w_t$ is the outcome at that round. For example if $(w_t,m_t)=(CD,(\frac{1}{2},\frac{1}{2}))$, then at round *t* both players randomized equally between C and D and (in consequence of this randomization) Player 1 cooperated and Player 2 defected.

We are interested in how the equilibria for the repeated game are affected by two factors: the information on which players base their choices at every round, and their time preferences.

We first considered information. Press and Dyson, like most of the existing literature, focus exclusively on games with *observable outcomes*, in which memory-one strategies could base their choice in round *t* only on the the outcome of the previous period, that is, on $w_{t-1}$. We depart from this by also considering games with *observable mixtures*, in which memory-one strategies may base their choice in round *t* also on the previous (mixed) strategy profile, $m_{t-1}$.

Second, we explored how equilibria are affected by players’ time preferences. To do this, we departed from the existing literature by considering the general case in which players may have different discount factors, which we denote with $\rho _i\in (0,1)$ ($i =1,2$). This implies that the present value of a unit payoff obtained after *t* rounds is $\delta _i^t$ with $\delta _i := \delta \rho _i$. We will show that the STG differs from the PD in that the set of cooperative equilibria for the repeated game only depends on the discount factor of Player 2. In particular, any cooperative equilibrium that exists for two equally patient players ($\delta _1=\delta _2>0$) would also exist if Player 1 were replaced by a myopic player who maximizes the expected payoff at the current round ($\delta _1 = 0$). Below we shall see that this provides a theoretical foundation for our experiment.

**Figure 7:**
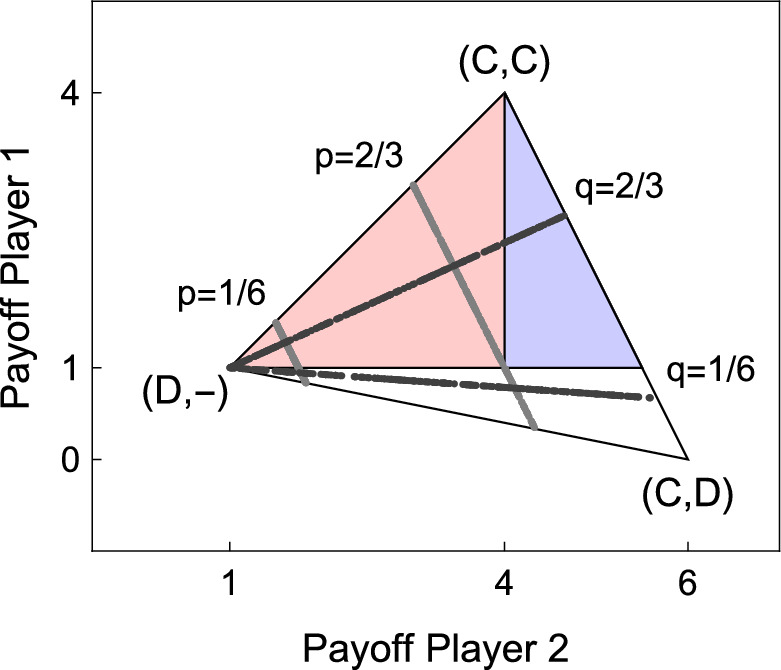
Players’ strategies in the payoff space. For each player, we fixed an unconditional strategy, and we let it play with 1000 randomly generated memory one strategies. Black dots are possible payoffs profiles when Player 2 sets $q=\frac{2}{3}$ or $q=\frac{1}{6}$. Gray dots are payoff profiles when Player 1 sets $p=\frac{2}{3}$ or $p=\frac{1}{6}$.

As we anticipated in “Introduction”, ExS for Player 2 assume a particularly simple form: At every round, Player 2 plays C with a fixed probability *q*, which, in the stage game, is large enough to make C the best reply for Player 1. Simple algebra suffices in showing that this probability needs to be larger than $q^S:=\frac{P-S}{R-S}$. In Fig. 7 each black dot represents the payoff profile that can be attained by the two players when Player 2 plays an unconditional strategy ($q=\frac{2}{3}$ or $q=\frac{1}{6}$), and Player 1 plays a randomly chosen memory-one strategy. Note that $q=\frac{2}{3}>q^S$ is an ExS (as the best reply of Player 1 would be full cooperation, and the resulting payoff profile would be in the blue area) while $q = \frac{1}{6}<q^S$ is not (as the best reply of Player 1 in this case would be to always defect, and both players would get the mutual defection payoff).

We investigated under which conditions ExS strategies can sustain an equilibrium, assuming that Player 1 is constrained to use memory-one strategies, both in an observable outcome and in an observable mixture setting. We denote with $\Pi = (\Pi _1,\Pi _2)$ a payoff profile for the repeated game, in which $\Pi _i$ is the expected payoff for player *i*. The set of payoff profiles on the Pareto frontier is *F*, and $\bar{F}$ is the subset of efficient profiles in which both players earn more than *P* (the dark blue segment in Fig. 1b). Finally, we denote with $\mathcal {E}_W$ and $\mathcal {E}_M$ the sets of payoff profiles that can be sustained as NE in games with observable outcomes and with observable mixtures, respectively.

### Main results

We first state a very general result that has a relatively simple proof (all the proofs are reported in “Supplementary Information”). With observable outcomes, if Player 1 is restricted to use memory-one strategies,the only efficient payoff profile that can be sustained in equilibrium is (*R*, *R*), implying that there are no extortionate equilibria on the Pareto frontier:

#### Proposition 1

*If Player 1 is constrained to memory-one strategies with observable outcomes, then*$\bar{F} \cap \mathcal {E}_W = (R,R)$.

The previous result is very general because it puts no restriction on the complexity of the strategy that Player 2 may use. However, it leaves open the possibility that there may be extortionate equilibria that do not lie on the Pareto frontier. While it can be proved that this is not the case, for our experiment it is sufficient to show that this cannot happen when Player 2 is restricted to *unconditional* strategies. To prove this result, we need a further piece of notation. Let *B* be the set of payoff profiles such that Player 1’s payoff is *P*, and Player 2’s payoff is at most *R*:

1 $$\begin{aligned} B = \{ (\Pi _1,\Pi _2) : \Pi _1=P \wedge P \le \Pi _2 \le R \} \end{aligned}$$

This set corresponds to the red segment in Fig. 1**b**. Our next Proposition shows that, if Player 1 is constrained to memory-one strategies and Player 2 is constrained to unconditional strategies, there is no equilibrium in which Player 2 obtains more than *R*, so that no extortion is possible in equilibrium:

#### Proposition 2

*If Player 1 is constrained to memory-one strategies with observable outcomes, and Player 2 is constrained to unconditional strategies, then*$\mathcal {E}_W= (R,R) \cup B$, *for any*$\delta _1 \in [0,1]$*and for*$\delta _2 \ge \frac{T-R}{T-P}$.

Our next proposition shows that in a game with observable mixtures, all the payoff profiles on the Pareto frontier in which Player 1 obtains more than *P* can be sustained in equilibrium, if Player 2 is sufficiently patient.

#### Proposition 3

*If Player 1 is constrained to memory-one strategies with observable mixtures, for any*$\delta _1 \in [0,1]$*and for*$\frac{T-R}{R-P}\le \delta _2 < 1$, $\bar{F}\subset \mathcal {E}_M$.

Note that in Propositions 2 and 3 no restriction is placed Player 1’s discount factor. Because of this, our results can be adapted to a setting similar to the one we used in our experiment, in which Players 2 are matched at every round with a new Player 1. To see this, suppose that the interaction takes place between individuals belonging to two populations, one of Players 1, and one of Players 2. At every round, each Player 2 is randomly matched with a different Player 1, and they play a single round of the STG. After each round the game continues with a probability $\delta \in (0,1)$ and players are assumed to be patient ($\rho _1=\rho _2=1$), so their payoff is the expected value of the strategy they choose, given $\delta$. We shall refer to this game as the *stranger* setting, to distinguish it from the one we discussed in the previous paragraph in which the same players interact repeatedly. We dub the latter *partner* setting.

Before playing, each Player 1 can observe how the current Player 2 behaved in the previous round. Player 2 is given no information about the past behavior of Player 1. This puts a restriction on the type of strategies that the players can employ. Players 2 are constrained to use *unconditional* strategies. Players 1 are constrained to use a subset of the set of memory-one strategies that are usually referred to as *reactive strategies*^41^. These strategies base the choice in the current round only on the other player’s behavior in the previous round while, by contrast, a general memory-one strategy bases its current choice on the outcome of the previous round, that is on the choice made by both players. As in the partner setting, we considered both the case in which Player 1 can observe the action (either C or D) played by Player 2 in the previous round, and the case in which she can also observe the mixed strategy previously chosen by Player 2. This model is in the spirit of^42–45^, where one long-lived Player 2 interacts repeatedly with a series of short-lived Players 1, and where each Player 1 can only observe how Player 2 behaved in the previous round.

Let $\hat{\mathcal {E}}_{W}$ and $\hat{\mathcal {E}}_{M}$ be the set of payoff profiles that can be sustained in equilibrium when the repeated STG is played among strangers,with observable outcomes and observable mixtures, respectively. The next proposition contains two results. First, when only the outcomes are observed, if a payoff profile can be sustained as a NE in the stranger setting, then it is also sustainable as an equilibrium in the partner setting. This is important, because we already know (from Proposition 2) that in the partner setting no extortion can take place in equilibrium if only outcomes are observed. Second, Proposition 4 says that, just like in the partner setting (see Proposition 3), when mixtures are observable there are efficient equilibria in which Player 2 obtains more than the mutual cooperation payoff.

#### Proposition 4

*Let Players 1 be constrained to reactive strategies and Players 2 to unconditional strategies. Then (a)*$\hat{\mathcal {E}}_{W} \subset \mathcal {E}_W$*and (b)*$\bar{F} \subset \hat{\mathcal {E}}_M$.

Our two experimental predictions are an immediate consequence of this proposition.

In our experiment, payoffs’ values are as shown in Fig. 1**a**. This implies that, in the Mixture treatment, extortionate equilibria exist for any $q\ge \frac{1}{4}$, as long as $\delta _2\ge \frac{2}{3}$. In the Outcome treatment, cooperative equilibria exist whenever $\delta _2 \ge \frac{2}{3(1-p_D)}$ (see Condition 11 in Supplementary Information), where $p_D$ is the probability with which Player 1 plays C when she observes D. Since it must be $\delta _2\le 1$, this implies $p_D \le \frac{1}{3}$. Thus, a cooperative equilibrium exists only if Player 1 punishes deviations by playing C with a probability smaller than $\frac{1}{3}$. This justifies our division between TfT and Forgiving strategies for Player 1.

### Experiment: participants and procedure

The experiment was run at the Cognitive and Experimental Economics Laboratory (CEEL, University of Trento, Italy), with a total of 112 subjects (54 for the Outcome treatments and 58 for the Mixture treatments), all of which were students at the University of Trento. The study was conducted using Z-tree^46^. Instructions were given neutrally, and actions were labeled as “Left” and “Right” for Player 2, and “Top” and “ Bottom” for Player 1.

The experiment was divided into two parts. In the first part (part A), subjects interacted directly with each other. In both treatments, subjects were randomly matched to play the STG for a minimum of 20 periods. To limit the end-game effect, a coin toss ensued at the end of every period after the 20th, to determine the ending of the game. At every round after the first one, Player 1 was informed of Player 2’s previous behavior. In the Mixture treatment, we provided information about the overall frequency with which each action was chosen. In the Outcome treatment, we gave only information about the last action taken by Player 2, thus forcing Player 1 to use a memory-one strategy.

In the second part (part B) we asked participants to submit their strategies in the form of simple computer programs. In both treatments, a strategy for Player 2 was a probability with which the computer had to play C in every period of the game. The set of available strategies was $\{0, 0.25, 0.5, 0.75, 1\}$. For example, if Player 2 chose the strategy 0.75, the computer would play C with probability 0.75 in every period of the game. The outcome of the strategy (either C or D), together with the action chosen by Player 1, would then determine Player 2’s payoff for that period.

A strategy for Player 1 was a *plan of behavior*: it specified one action (either C or D) to be played in the first period of the game, and a set of actions (one action for each possible contingency) to be played afterwards. In the Mixture treatment, a strategy for Player 1 had to specify, for each possible mixed strategy that Player 2 could have chosen, whether to play C or D. For example, a subject could instruct the computer to play C when the strategy played by the other player was 0.5, and D otherwise. In this case, the computer would play C in all and only the rounds in which it was matched with a Player 2 who choose to play C with probability 0.5. In the Outcome treatment, a strategy for Player 1 had to specify the action to take for each possible *outcome* (i.e. either C or D) of Player 2’s strategy. For example, if a subject instructed the computer to play C after C, and D after D, then the computer would play C in every period in which it encountered a Player 2 who played C in the previous period, independently from the mixed strategy chosen by Player 2.

For each treatment, we collected all the strategies and let the computer play them for 20 periods. That is, in every period of the game the computer would match each Player1 with a different Player 2. In every period after the first one, the computer would check the previous behavior of Player 2, and it would select the corresponding action of Player 1 according to the plan of behavior that Player 1 submitted at the beginning of the game. Similarly, the computer would select an action for Player 2 according with the strategy that Player 2 submitted at the beginning of the game, and would thus determine the outcome of that round.

The experiment lasted about 60 min. Payoffs were expressed in experimental monetary units (EMS).

### Ethical approval

We obtained a written informed consent from all our participants. All experimental procedures were in accordance with the Declaration of Helsinki, and with the General Data Protection Regulation (GDPR) of the European Union. The study was approved by the Comitato etico per la ricerca (Ethics Commitee for Research, CER) of the University of Trento.

## Supplementary Information

Supplementary Information 1.
