# Supplementary material for: Human players manage to extort more than the mutual cooperation payoff in repeated social dilemmas
Source: Sci Rep. 2021 Aug 19;11:16820. doi: 10.1038/s41598-021-96061-9 (PMC8377025; doi:10.1038/s41598-021-96061-9)
Supplement: Supplementary file 1 — Supplementary Information 1. [file 41598_2021_96061_MOESM1_ESM.pdf]

Human players manage to extort more than the mutual cooperation payoff in repeated social dilemmas - Supplementary Information

Chiara D'Arcangelo[1,*], Luciano Andreozzi[2], and Marco Faillo[2]

[1]Università degli studi G. D'Annunzio Chieti - Pescara, Dipartimento di Economia, Pescara, 65127, Italy
[2]Università di Trento, Dipartimento di Economia, Trento, 38100, Italy
[*]chiara.darcangelo@unich.it

1 Theoretical Methods

We start with the standard, repeated game setting that we called the *partner setting* in the Method. The TG is played repeatedly between the same Player 1 and Player 2 with continuation probability $\delta \in (0,1)$. Player i discounts future payoffs with a discount factor $\delta_i \in [0,1]$. As a consequence, a unit payoff after t rounds has a present value $(\rho_i \delta)^t$. We let $\delta_i := \rho_i \delta$.

We denote with $W = \{CC, CD, DC, DD\}$ the set of possible outcomes of the game, where w is a generic element of W. p and q are players' mixed strategies for the stage game, and $m = (p,q)$ is a generic mixed strategy profile. For the repeated game, (w_t, m_t) is the *mixture outcome* of the game, where m_t is the mixed strategy profile chosen at round t and w_t is the outcome at that round. Let $h_t = \{(w_0, m_0)....(w_{t-1}, m_{t-1})\}$ be a history of play up to round t. H_t is the set of all possible histories up to round t and $H = \cup_{t=0...\infty} H_t$ is the set of all possible histories. A *strategy* for player i is a map from the set of possible histories to his strategy set: $s_i : H \to M_i := \{[0,1]\}$. Let $s_i(h_t)$ be player i's continuation strategy after h_t. We need the following definition:

Definition 1 (Memory-one strategies)**.** *Let h and h' be two histories, and let $h(t-1) = (w_{t-1}, m_{t-1})$ and $h'(t-1) = (w'_{t-1}, m'_{t-1})$ be their components at period $t-1$. A strategy s_i is:*
(i) memory-one with observable mixtures *if $m_{t-1} = m'_{t-1}$ implies $s_i(h_t) = s_i(h'_t)$;*
(ii) memory-one with observable outcomes *if $w_{t-1} = w'_{t-1}$ implies $s_i(h_t) = s_i(h'_t)$;*
(iii) unconditional *if $s_i(h_t) = s_i(h'_t)$ $\forall$ h_t and h'_t.*

The stage game payoff of player i when the outcome is w is $\pi_i(w)$. Following the standard notation for the PD, in the stage game we denote with T the temptation payoff, with S the sucker's payoff, with R the reward for mutual cooperation, and with P the punishment for mutual defection. In the repeated game, $\Pi_i = \Pi_i(s_1, s_2)$ is the average payoff for player i when the strategy profile is (s_1, s_2). F is the Pareto frontier, and $\bar{F}$ is the part of the Pareto frontier that is individually rational.

Finally, we denote by $\mathscr{E}_W$ the set of payoff profiles that can be sustained as NE with observable outcomes (not necessarily SPNE), and by $\mathscr{E}_M$ the set of payoff profiles that can be sustained as NE with observable mixtures (not necessarily SPNE).

The following Propositions describe the sets $\mathscr{E}_W$ and $\mathscr{E}_M$.

Proposition 1 If Player 1 is constrained to memory-one strategies with observable outcomes, then $\bar{F} \cap \mathscr{E}_W = (R,R)$.

Proof. We have to show that, if an equilibrium payoff profile is *on* the Pareto frontier, F, then the payoff profile must be (R,R). Following[1], let $v_w(t)$ be the probability that, given a generic strategy profile (s_1, s_2), the outcome w is observed in period t , and let v_w be the average probability that the outcome w is observed, that is:

$$v_w = (1-\delta) \sum_{t=0}^{\infty} \delta^t v_w(t) \tag{1}$$

In order for a payoff profile to lie on the Pareto frontier, the probability to go to states DC and DD must be zero, that is, it must be $v_{DC} = v_{DD} = 0$. Following[1] and[2], it is easy to show that this is the case only if: i) $v_{CC} = 1$, or ii) $v_{CD} = 1$, or iii) $p_0 = p_{CC} = p_{CD} = 1$. The first condition implies that only CC is observed on the path of play, resulting in the payoff profile (R,R). The second condition implies that only CD is observed on the path of play, resulting in the payoff profile (S,T). This can never be an equilibrium, since Player 1's payoff is smaller than P. Also (iii) can never hold in a Nash equilibrium. To see why, just note that, if $p_0 = p_{CD} = 1$, the best reply of Player 2 is to play D at each round, as this would give him T, the maximum payoff he can get. However, this implies that only CD is observed, and we know that $v_{CD} = 1$ cannot be an equilibrium. It follows that the only way for a payoff profile to lie on the Pareto frontier is when condition i) holds, implying that the only payoff profile that can be reached is (R,R). □

Proposition 2 If Player 1 is constrained to memory-one strategies with observable outcomes, and Player 2 is constrained to unconditional strategies, then $\mathscr{E}_W = (R,R) \cup B$, for any $\delta_1 \in [0,1]$ and for $\delta_2 \geq \frac{T-R}{T-P}$, where

$$B = \{(\Pi_1, \Pi_2): \quad \Pi_1 = P \quad \wedge \quad P \leq \Pi_2 \leq R\}$$

Proof. We characterize the payoff profiles that can be sustained in a Nash equilibrium, non necessary subgame perfect, in which Player 1 can use a memory one strategy of the form $\mathbf{p} = (p_0, p_{CC}, p_{CD}, p_{DC}, p_{DD})$, and Player 2 uses an unconditional strategy of the form $q_0 = q_w = q$ for every outcome w and some $q \in [0,1]$.

Let $\Pi_i(w)$ be the total average payoff of player i if the outcome of the current round is w. Following[3], we can write it as:

$$\Pi_i(w) = (1-\delta_i)\pi_i(w) + \delta_i \gamma_i(w) \tag{2}$$

where $\gamma_i(w)$ is the total average payoff following outcome w, that is:

$$\gamma_i(w) = p_w q \Pi_i(CC) + p_w(1-q)\Pi_i(CD) + (1-p_w)q\Pi_i(DC) + (1-p_w)(1-q)\Pi_i(DD) \tag{3}$$

Thus, the total average payoff of player i if the current round's outcome is w is a weighted average (with weights $1-\delta_i$ and δ_i) of the payoff obtained in w, $\pi_i(w)$, and the expected payoff after w, $\gamma_i(w)$. We have then a system of four equations, which we can solve for each $\Pi_i(w)$. This allows us to compute the total average payoff of player i at the beginning of the game as:

$$\Pi_i = p_0 q \Pi_i(CC) + (1-q)p_0\Pi_i(CD) + (1-p_0)q\Pi_i(DC) + (1-p_0)(1-q)\Pi_i(DD) \tag{4}$$

Consider first Player 1. The expected payoff if he plays C after the outcome w (that is, if he sets $p_w = 1$) is $\Pi_1^C := q\Pi_1(CC) + (1-q)\Pi_1(CD)$, while if he plays D after w ($p_w = 0$) his payoff is $\Pi_1^D := q\Pi_1(DC) + (1-q)\Pi_1(DD)$. We can then write the difference between the continuation payoffs of Player 1 after two different outcomes as:

$$\begin{aligned}\gamma_1(w) - \gamma_1(w') = &(p_w - p_{w'})q\Pi_1(CC) + (p_w - p_{w'})(1-q)\Pi_1(CD) - \\ &- (p_w - p_{w'})q\Pi_1(DC) - (p_w - p_{w'})(1-q)\Pi_1(DD) = \\ &= (p_w - p_{w'})(q(\Pi_1(CC) - \Pi_1(DC)) + (1-q)(\Pi_1(CD) - \Pi_1(DD))) = \\ &= (p_w - p_{w'})(\Pi_1^C - \Pi_1^D)\end{aligned} \tag{5}$$

That is, the difference between the average payoffs given two different outcomes that might happen in the current round is:

$$\begin{aligned}\Pi_1(w) - \Pi_1(w') &= (1-\delta_1)(\pi_1(w) - \pi_1(w')) + \delta_1(\gamma_i(w) - \gamma_i(w')) = \\ &= (1-\delta_1)(\pi_1(w) - \pi_1(w')) + \delta_1(p_w - p_{w'})(\Pi_1^C - \Pi_1^D)\end{aligned} \tag{6}$$

At the beginning of the game, and after each outcome w that is observed on the path of play, Player 1 will play C if $\Pi_1^C > \Pi_1^D$. Equation 6 implies that we can write the difference $\Pi_1^C - \Pi_1^D$ as:

$$\begin{aligned}\Pi_1^C - \Pi_1^D =&(q\Pi_1(CC) + (1-q)\Pi_1(CD)) - (q\Pi_1(DC) + (1-q)\Pi_1(DD)) = \\ =&q(\Pi_1(CC) - \Pi_1(DC)) + (1-q)(\Pi_1(CD) - \Pi_1(DD)) = \\ =&q(1-\delta_1)(\pi_1(CC) - \pi_1(DC) + \delta_1(p_{CC} - p_{DC})(\Pi_1^C - \Pi_1^D) + \\ &+(1-q)(1-\delta_1)(\pi_1(DD) - \pi_1(CD) + \delta_1(p_{CD} - p_{DD})(\Pi_1^C - \Pi_1^D))\end{aligned} \tag{7}$$

which implies:

$$\Pi_1^C - \Pi_1^D = \frac{\delta_1(q(R-S) - (P-S))}{1 - \delta_1(q(p_{CC} - p_{DC}) + (1-q)(p_{CD} - p_{DD}))} \tag{8}$$

That, is $\Pi_1^C > \Pi_1^D$ if $q > q^S := \frac{P-S}{R-S}$, and this implies the following:

$$\begin{cases} p_0 = p_w = 1 & \text{if} \quad \Pi_1^C > \Pi_1^D \quad \iff \quad q^S < q \leq 1 \\ p_0 = p_w = 0 & \text{if} \quad \Pi_1^C < \Pi_1^D \quad \iff \quad 0 \leq q < q^S \\ p_0 \in [0,1] \quad \wedge \quad p_w \in [0,1] & \text{if} \quad \Pi_1^C = \Pi_1^D \quad \iff \quad q = q^S \end{cases} \tag{9}$$

Condition 9 imposes a constraint only after those outcomes that are observed with positive probability, allowing Player 1 to choose any value of p_w for all outcomes w which are never observed on the path of play. Clearly, if $q \in \{0,1\}$, two outcomes can never be observed, and so these cases need to be discussed separately. This gives us five cases to consider.

Case 1 If $q = 1$, Condition 9 implies that Player 1 sets $p_0 = 1$. Thus, the path of play starts at CC, implying that this outcome is observed. Invoking again Condition 9 we get $p_{CC} = 1$. Since $q = 1$, this implies that CC is also the only outcome observed and, again from 9, Player 1 can set the other probabilities arbitrarily. Consider now Player 2. Since only the outcome CC is observed in equilibrium, his payoff will be $\Pi_2^{eq} = \Pi_2(CC) = R$. Using the one-step deviation property, the payoff of Player 2 if he deviates by playing D in the current round and then goes back to $q = 1$ is:

$$\Pi_2^{dev} = \Pi_2(CD) = (1-\delta_2)T + \delta_2(p_{CD}R + (1-p_{CD})\frac{P(1-\delta_2) + R\delta_2 p_{DC}}{1-\delta_2(1-p_{DC})}) \tag{10}$$

Player2 will set $q = 1$ if $\Pi_2^{eq} = R \geq \Pi_2^{dev}$, that is:

$$1 \geq \delta_2 \geq \hat{\delta} = \frac{T-R}{T-P-p_{CD}(R-P)-p_{DC}(T-R)} \tag{11}$$

Note that $\hat{\delta}$ is minimized when $p_{CD} = p_{DC} = 0$, and hence the condition becomes $\delta_2 \geq \frac{T-R}{T-P}$.

Case 2 If $q^S < q < 1$, like in the previous case, condition 9 implies that Player 1 will set $p_0 = 1$. Since $q^S < q < 1$ and $p_0 = 1$, both CC and CD have a positive probability to be observed. Condition 9 then implies that $p_{CC} = p_{CD} = 1$. However, from the proof of Proposition 1, we know that there is no NE in which Player 1 sets $p_0 = p_{CD} = 1$.

Case 3 If $0 < q < q^S$, condition 9 implies that Player 1 will set $p_0 = 0$. Similarly to the previous case, both DC and DD have a positive probability to be observed, and hence condition 9 implies $p_{DC} = p_{DD} = 0$, that is, on the path of play Player 1 always plays D. This in turn implies that the payoff of Player 2 is P, independently from which strategy he chooses. Since he is indifferent, he can choose any $q \in (0, q^S)$. The resulting payoff profile is (P, P), and it is independent from the players' discount factors.

Case 4 If $q = 0$, condition 9 implies that Player 1 sets $p_0 = 0$. Thus, the path of play starts at DD, implying that this outcome is observed, and hence $p_{DD} = 0$. Since $q = 0$ this implies that DD is also the only outcome observed and, again from 9, Player 1 can set the other probabilities arbitrarily. Consider now Player 2. If he sets $q = 0$, the path of play starts at DD, and, given $p_0 = p_{DD} = 0$, from System 2 we get $\Pi_2^{eq} = \Pi_2(DD) = P$. If Player 2 deviates by playing C in the current round and then goes back to $q = 0$, his payoff is:

$$\Pi_2^{dev} = \Pi_2(DC) = P + p_{DC}(T-P)\frac{\delta_2(1-\delta_2)}{1-\delta_2 p_{CD}} \tag{12}$$

Then, it is optimal to set $q = 0$ whenever $P \geq \Pi_2^{dev}$, which holds only if $p_{DC} = 0$. As in the previous case, the resulting equilibrium payoff profile is (P, P),

Case 5 If $q = q^S$, Player 2 is always mixing, so he must be indifferent between C and D. This implies that, after any w that is observed, it must hold:

$$p_w\Pi_2(CC) + (1-p_w)\Pi_2(DC) = p_w\Pi_2(CD) + (1-p_w)\Pi_2(DD) \tag{13}$$

where the lhs is the payoff if Player 2 chooses C while the rhs is the payoff if he chooses D. We can rearrange condition 13 as:

$$p_w(\Pi_2(CC) - \Pi_2(CD) + \Pi_2(DD) - \Pi_2(DC)) = \Pi_2(DD) - \Pi_2(DC) \tag{14}$$

This condition is satisfied only if one of the following holds true:

$$p_w = \frac{\Pi_2(DD) - \Pi_2(DC)}{\Pi_2(CC) - \Pi_2(CD) + \Pi_2(DD) - \Pi_2(DC)} = \hat{p} \quad \text{for some} \quad \hat{p} \in [0,1] \tag{15a}$$

$$\Pi_2(DD) - \Pi_2(DC) = 0 \quad \text{and} \quad \Pi_2(CC) - \Pi_1(CD) = 0 \tag{15b}$$

Consider first Condition 15a. It implies that Player 1 plays C with the same probability $\hat{p}$ after every outcome that is observed. Three cases are possible:

- Player 1 is mixing and all outcomes are observed on the path of play. This requires that for every w, $p_0 = p_w = \hat{p} \in (0,1)$. Given $p_w = \hat{p}$, Equation 2 implies that for every pair of outcomes w and w', $\Pi_2(w) - \Pi_2(w') = (1-\delta_2)(\pi_2(w) - \pi_2(w')$. It follows that $\Pi_2(CC) - \Pi_2(CD) = (1-\delta_2)(R-T) < 0$, and $\Pi_2(DD) - \Pi_2(DC) = (1-\delta_2)(P-P) = 0$. The latter equation implies that $\hat{p} = 0$, which contradicts the hypothesis that Player 1 is mixing. So no equilibrium corresponds to this case.

- Player 1 always plays C and only the outcomes CC and CD are observed. This requires that $p_0 = p_{CC} = p_{CD} = 1$. However, from the proof of Proposition 1 we know that this cannot be true in equilibrium.
- Player 1 always plays D and only the outcomes DC and DD can be observed, which requires that $p_0 = p_{DC} = p_{DD} = \hat{p} = 0$. This is an equilibrium with payoff profile (P,P).

Consider now Condition 15b. It implies that Player 2 is always indifferent, independently from the probability with which Player 1 plays C *in the current round*. To see this, note that the payoff of Player 2 is $\Pi_2(CC) = \Pi_2(CD) := X$ if Player 1 chooses C today, and is $\Pi_2(DD) = \Pi_2(DC) := Y$ if Player 1 chooses D today, independently from the strategy of Player 2. If a strategy profile satisfies Equations 17, it forms a *belief free equilibrium* as in[4], and we call such a strategy a belief-free strategy. From Equations 2 we have:

$$\Pi_2(w) = (1-\delta_2)\pi_2(w) + \delta_2(p_w X + (1-p_w)Y) \tag{16}$$

Solving for each p_w we get:

$$p_w = \frac{\Pi_2(w) - ((1-\delta_2)\pi_2(w) + \delta_2 Y)}{\delta_2(X-Y)} \tag{17}$$

where $\pi_2(w)$ is the stage game payoff of Player 2 if the current round's outcome is w, $\Pi_2(w) = X$ if $w \in \{CC, CD\}$, and $\Pi_2(w) = Y$ if $w \in \{DC, DD\}$. The total average payoff of Player 2 will then depend on the probability with which Player 1 plays C at the beginning of the game, that is $\Pi_2 = p_0 X + (1-p_0)Y$.

In order for probabilities in Equations 17 to be between 0 and 1, one needs $R \geq X > Y \geq P$ and $\delta_2 > \max\{\frac{Y-P}{X-P}, \frac{T-X}{T-Y}\} = \hat{\delta}$. To see this, note that Equations 17 imply $p_{CC} = \frac{X-((1-\delta_2)R+\delta_2 Y)}{\delta_2(X-Y)}$, $p_{DD} = p_{DC} = \frac{Y-((1-\delta_2)P+\delta_2 Y)}{\delta_2(X-Y)} = \frac{(1-\delta_2)(Y-P)}{\delta_2(X-Y)}$, and $p_{CD} = \frac{X-((1-\delta_2)T+\delta_2 Y)}{\delta_2(X-Y)}$. To have $1 \geq p_{DD} \geq 0$ one needs: $X > Y$, $Y \geq P$ and $\delta_2 \geq \frac{Y-P}{X-P}$. Given that, to have $1 \geq p_{CC} \geq 0$, one needs $X \leq R$, and to have $1 \geq p_{CD} \geq 0$, one needs $\delta_2 \geq \frac{T-X}{T-Y}$. That is, for $\delta_2 \geq \hat{\delta}$, Player 1 can set the payoff of Player 2 anywhere between P and R by choosing proper values of X, Y, and p_0. Next, we note that $\frac{T-X}{T-Y}$ is minimized when $X = R$ and $Y = P$, while $\frac{Y-P}{X-P}$ is minimized when $Y = P$. Thus, whenever $\delta_2 > \frac{T-R}{T-P}$, there is a belief free strategy that Player 1 can use that is able to set the payoff of Player 2 anywhere between R and P, by setting $X = R$ and $Y = P$, and choosing a proper value of p_0.

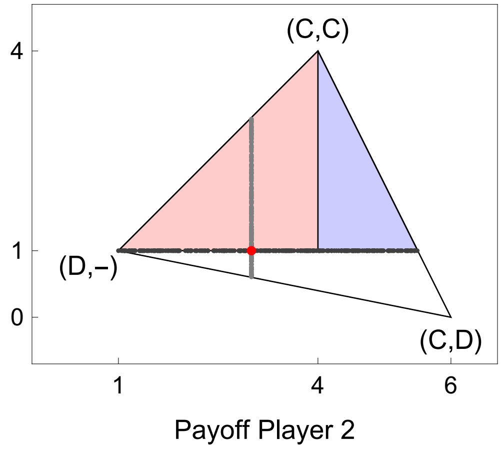

Figure 1. An example of mixed equilibrium. To obtain the dots, we fix a strategy for each player, and we let it play with 1000 random generated memory one strategies of the opponent. Player 2 (black dots) uses an unconditional strategy and sets $q = q^S = \frac{1}{4}$. Player 1 (grey dots) uses a belief free (equalizer) strategy, and sets $p_0 = 1, p_{CC} = \frac{3}{4}, p_{CD} = \frac{1}{4}, p_{DC} = p_{DD} = 0$. $R = 4, P = 1, S = 0, T = 6$, $\delta = \frac{9}{10}$. $R = 4, P = 1, S = 0, T = 6$. The red dot corresponds to the Nash equilibrium profile.

To recap, if Player 1 is constrained to use a memory one strategy with observable outcomes, there can only be three types of equilibria:

- *cooperative*: $p_0 = p_{CC} = q = 1$, with payoff profile (R,R), for $\delta_2 \geq \frac{T-R}{T-P}$
- *non cooperative*: $p_0 = p_{DC} = p_{DD} = 0$ and $0 \leq q \leq q^S$, with payoff profile (P,P)
- *mixed*: $q = q^S$ and $\mathbf{p}$ is a belief free strategy, with payoff profile (P, Π_2), with $P \leq \Pi_2 \leq R$ and $\delta_2 \geq \frac{T-R}{T-P}$.

That is, the set of Nash equilibrium payoff profiles is $\mathscr{E}_W = (R,R) \cup B$.

□

Proposition 3 For the repeated TG with observable mixtures, for any $\delta_1 \in [0,1]$ and for $\frac{T-R}{R-P} \le \delta_2 < 1$, $\bar{F} \subset \mathscr{E}_M$.

Proof. At each round each player observes the mixed strategy profile chosen in the previous round $m \in M$ and the previous outcome $w \in O$. We represent memory-one strategy for Player 1 as $(p_0, P(w,m))$, where p_0 is the probability with which she plays C at the first round and $P(w,m) \in [0,1]$ is the probability with which she plays C when the mixture-outcome of the previous round is (w,m). Player 2's strategy $(q_0, Q(w,m))$ is defined analogously.

We prove the existence of efficent exortionary equilibria for this game in the following way.

First, consider an *unconditional* strategy for player 2: $q_0 = Q(w,m) = \bar{q}$ for some $\bar{q} \in [0,1]$. This strategy dictates that player 2 plays C with a fixed probability $\bar{q}$ regardless of the mixture outcome in the previous round. We shall represent such a strategy as $Q_{\bar{q}}$.

For Player 2, we consider a memory-one strategy in the form: $p_0 = 1$ and

$$P(w,(p,q)) = \begin{cases} 1, & \text{if} \quad q \ge \underline{q} \\ 0, & \text{if} \quad q < \underline{q} \end{cases} \tag{18}$$

This is a memory-one strategy whereby Player 1 only conditions on the probability with which Player 2 choose C in the previous round, but not on the outcome of the game. This strategy requires that Player 1 plays C if and only if in the previous round Player 2 has played C with a probability no smaller than $\underline{q}$. We shall represent this strategy as $P_{\underline{q}}$.

Our proof consists in showing that (P_{q^*}, Q_{q^*}) is a NE for the repeated TG with observable mixtures if $q^* > \frac{P-S}{R-S}$ and $\frac{(T-R)q^*}{(T-P)-(T-R)q^*} \le \delta_2 < 1$. First note that, since Player 2 uses mixed strategy q^* at every round, the observed mixture outcome is $((p,q^*),w)$ for some (not necessarily constant) $p \in [0,1]$ and $w \in W$. Second, since $\underline{q} = \bar{q} = q^*$, Player 1 plays C at every round and hence the expected payoff profile is

$$\pi_1^* := \pi_1(P_{q^*}, Q_{q^*}) = Rq^* + S(1-q^*) \tag{19}$$
$$\pi_2^* := \pi_2(P_{q^*}, Q_{q^*}) = Rq^* + T(1-q^*) \tag{20}$$

Consider first Player 1. Using the one-shot deviation property, the equilibrium condition is

$$Rq^* + S(1-q^*) \ge (1-\delta_1)P + \delta_1(Rq^* + S(1-q^*))$$

The l.h.s. of this inequality is the equilibrium payoff, the r.h.s. is the payoff of Player 1 if he switches from C to D in the current round and then resumes her equilibrium play. Rearranging we obtain $q^* \ge \frac{P-S}{R-S}$.

Consider now Player 2. To use the one-step deviation property, suppose that in the current round Player 2 chooses a probability $q \ne q^*$ and reverts to her equilibrium strategy for the rest of the game. If $q > q^*$, then Player 1 will play C in the following round. So the equilibrium condition is:

$$T(1-q^*) + Rq^* \ge (1-\delta_2)(Rq + T(1-q)) + \delta_2(Rq^* + T(1-q^*))$$

which is always true if $q > q^*$. If instead $q < q^*$, Player 1 will play D in the following round (when Player 2 play q^*) and following that the equilibrium play resumes. The equilibrium condition then requires that

$$T(1-q^*) + Rq^* \ge (1-\delta_2)(Rq + T(1-q)) + \delta_2(1-\delta_2)P + \delta_2^2(Rq^* + T(1-q^*))$$

Solving for δ_2 we get

$$\frac{(T-R)(q^*-q)}{(T-P)-(T-R)q^*} \le \delta_2 < 1$$

The value of this threshold for δ_2 is maximized when $q = 0$ and $q^* = 1$, which gives us the second condition of our proposition. □

Proposition 4 Let Players 1 be constrained to reactive strategies and Players 2 to unconditional strategies. Then (a) $\hat{\mathscr{E}}_W \subset \mathscr{E}_W$ and (b) $\bar{F} \subset \hat{\mathscr{E}}_M$.

Proof. **Proof of (a)** We prove this proposition in two steps.

Step 1. Consider first a game played repeatedly by the same players, in which Player 1 uses a reactive strategy, and Player 2 uses an unconditional strategy. By following the proof of Proposition 2 with the assumptions that $p_{CC} = p_{DC} = p_C$ and $p_{CD} = p_{DD} = p_D$, it is easy to see that mutual cooperation is an equilibrium for $\delta_2 > \frac{T-R}{R-P}$ (since $p_{DC} = 1$). The non cooperative

equilibrium clearly remains, as this is the NE of the stage game. The mixed equilibrium requires that Player 2 sets $q = q^S$, and Player 1 uses a belief free strategy of the form:

$$p_{CC} = \frac{X - ((1-\delta_2)R + \delta_2 Y)}{\delta_2(X-Y)}; \quad p_{CD} = \frac{X - ((1-\delta_2)S + \delta_2 Y)}{\delta_2(X-Y)}; \quad p_{DC} = p_{DD} = \frac{(Y-P)}{\delta_2(X-Y)}$$

where X and Y are the payoffs of Player 2 if, in the current round, Player 1 plays C or D, respectively. Note that this strategy requires $p_{DC} = p_{DD}$. That is, it can never be reactive unless $p_{DC} = p_{DD} = 0$. This in turn implies that, if Player 1 uses reactive strategies, the only way he has to make the opponent indifferent is to always play D, resulting in the payoff profile (P,P).

Step 2. Consider now the game played by two populations. In the population of Player 2, a fraction x_j of players plays C w.p. q_j at every round. In the population of Player 1, a fraction y_j of the population plays the memory one strategy $\mathbf{p}_j$ (that is, they play C w.p. p_{wj} after outcome w, and w.p. p_{0j} in the first round). Since Player 1 is using a reactive strategy, we have $p_{CCj} = p_{DCj} = p_{Cj}$ and $p_{CDj} = p_{DDj} = p_{Dj}$.

Consider first Player 2. In the first period of the game, he faces one Player 1, randomly extracted from a population in which fraction y_j of players plays C w.p. p_{0j}. The probability that this Player 1 will play C is then:

$$\hat{p}_0 = \sum_j y_j p_{0j}$$

At any round after the first one, the probability that Player 1 plays C, given that the previous outcome was w, is:

$$\hat{p}_w = \sum_j y_j p_{wj}$$

Assuming that the composition of the population of Player 1 is fixed, we can then write the total average payoff of Player 2 as:

$$\Pi_2 = (1-\delta_2)[\hat{p}_0(qR + (1-q)T - P)] + \delta_2[(\hat{p}_C q + \hat{p}_D(1-q))(qR + (1-q)T - P)] + P \tag{21}$$

That is, playing against a population of memory one players is equivalent to play against a single memory one player whose strategy is:

$$\hat{p} = (\hat{p}_0, \hat{p}_C, \hat{p}_D) = (\sum_j y_j p_{0j}, \sum_j y_j p_{Cj}, \sum_j y_j p_{Dj})$$

Note that, if either $\hat{p}_w = 0$ or $\hat{p}_w = 1$, it must be that, for each strategy j s.t. $y_j > 0$, $p_{wj} = 1$ or $p_{wj} = 0$. This directly implies that the result from Proposition 1 still holds. That is, if the population of Players 1 is constrained to reactive strategies, the only profile on the Pareto frontier that can be sustained in a Nash equilibrium is (R,R), regardless of the strategy used by Player 2.

Consider now Player 1. In any round, he plays against a Player 2 randomly extracted from a population where a fraction y^t of players plays C w.p. q^j. His payoff can be computed as

$$\begin{aligned}\Pi_1 = (1-\delta)(p_0(\hat{q}R + (1-\hat{q})S - P)) + \delta(\hat{q}(p_C(Rp(C|C) + S(1-p(C|C)) + (1-p_C)P) + \\ + (1-\hat{q})(p_D(Rp(C|D) + S(1-p(C|D)) + (1-p_D)P))\end{aligned} \tag{22}$$

where

$$\hat{q} = \sum_j x_j q_j$$

is the probability that a Player 2 plays C in the first round, and

$$p(C|C) = \sum_j p(C|q_j)p(q_j|C) = \sum_j p(C|q_j)\frac{p(C|q_j)p(q_j)}{p(C)} = \sum_j q_j \frac{q_j x_j}{\hat{q}} \tag{23}$$

$$p(C|D) = \sum_j p(C|q_j)p(q_j|D) = \sum_j p(C|q_j)\frac{p(D|q_j)p(q_j)}{p(D)} = \sum_j q_j \frac{(1-q_j)x_j}{1-\hat{q}} \tag{24}$$

are the (updated) probabilities with which a Player 2 will play C after he played C or D, respectively.

Assume first that all Players 2 use the same strategy, that is, they play C w.p. q. In this case, we have that $p(C|C) = p(C|D) = \hat{q} = q$, and we can write:

$$\Pi_1 = (1-\delta)(p_0(\hat{q}R + (1-\hat{q})S - P)) + \delta((p_C\hat{q} + p_D(1-\hat{q})(\hat{q}R + (1-\hat{q})S - P) + P \tag{25}$$

Clearly, playing against a population of players choosing the same q_j is the same as playing against one Player 2 playing $q = \hat{q}$. That is, the equilibria are the same as those we have found in Step 1.

Assume now that Players 2 are using different q_j. In equilibrium, every q_j must be a best reply to $\hat{p}$, which implies that all q_j must give to Player 2 the same payoff against $\hat{p}$. However, from Step 1 we know that this is only possible if Player 1 always plays D, which implies that, if in equilibrium Players 2 are choosing different probabilities, then the resulting payoff profile can only be (P,P).

In turn, this implies that $\hat{\mathscr{E}}_W = \{(R,R),(P,P)\} \subset \mathscr{E}_W$. □

Proof. **Proof of(b)** With observable mixtures, we have to show that $\bar{F} \subset \hat{\mathscr{E}}_M$.

We consider a population of Player 2, where a fraction x_j plays C w.p. q_j at every round, and a population of Player 1, where a fraction y_j plays the memory one strategy $P^j = (p_0^j, P^j(w,(p,q))$. That is, they play C w.p. p_0^j in the first round, and w.p. $P^j(w,(p,q))$ if the previous mixture-outcome of the game was $(w,(p,q))$. Since Player 1 is using a reactive strategy, we have $P^j(CC,(p,q)) = P^j(DC,(p,q)) = P^j(C,q)$ and $P^j(CD,(p,q)) = P^j(DD,(p,q)) = P^j(D,q)$.

Note that, in the proof of Proposition 3 we showed that the profiles on the Pareto frontier can be sustained as a NE if Player 2 sets $q > \frac{P-S}{R-S}$ and Player 1 uses a strategy s.t. $p_0 = 1$ and

$$P(w,(p,q)) = \begin{cases} 1, & \text{if} \quad q \geq \underline{q} \\ 0, & \text{if} \quad q < \underline{q} \end{cases} \tag{26}$$

Note that this strategy implies $P(w,(p,q)) = P(q)\ \forall w$. That is, Player 1 can use this strategy also when he is restricted to reactive strategies. This in turn implies that there is an equilibrium where all Players 2 set $q^j = q^* > \frac{P-S}{R-S}$ and all Players 1 set $P^j(C,q) = P^j(D,q) = P(q)$ s.t. $\underline{q} = q^*$.

□

2 Strategies classification

2.1 Player 1 strategies

We classified Players 1 considering their behavior in periods 6-20.

In the Direct Response Method, we classified Players 1 starting from the data in Figure 6. In the Mixture treatment, we used the following procedure. Let q the probability of cooperation observed at round t.

Step 1: compute the maximum value of q above which a subject played D (*max*), and the minimum value after which he played C (*min*);

Step 2: compute $d = |max - min|$. Note that for a player who is consistent in using a step strategy it should be the case that $d = 0$. So we take d as an index of how much a player deviates from a step strategy;

Step 3a: if $d \geq 0.25$, we classify the subject as "other";

Step 3b: if $d < 0.25$, we classify the subject according to whether the average $a = \frac{max+min}{2}$ is in the intervals $[0, 0.25)$, $[0.25, 0.5)$, $[0.5, 0.75)$ and we denote each interval with its lower bound;

Step 3c: if a subject always played C (or D), we classify him according to the lowest (highest) value of q that he observed. Two subjects always played C. Since they never observed a frequency smaller than 0.25 ($min = 0.33$ and 0.43), we classify them in the interval $[0.25, 0.5)$.

In the Outcome treatment, we used the following procedure:

Step 1: compute the frequency with which a subject played C after observing C (p_C) and after observing D (p_D);

Step 2a: if $p_C = p_D = 0$, a subject is classified as AllD;

Step 2b: if $p_C = p_D = 1$, a subject is classified as AllC

Step 2c: if $p_C = 1$ and $p_D \leq 1/3$ (that is the maximum value that is able to sustain the cooperative equilibrium), a subject is classified as TfT

Step 2d: if $p_C = 1$ and $p_D > 1/3$, a subject is classified as Forgiving (Forg)

Step 2e: in all other cases, a subject is classified as "other".

With this classification, Table 1 shows the frequency and the performance of each strategy of Player 1. In the Mixture treatment, the most used strategy prescribe to play D if the observed frequency is smaller than .5, in both DRM and SM. However, in SM there is almost no difference between strategies' average payoffs', even if more tolerant players (that is, players with a lower threshold value) gets a slightly higher payoff than less tolerant ones. This tendency is more pronounced in DRM, as strategies' payoff is increasing in the "tolerance level" of the player. In the Outcome treatments, the most used strategy is TfT, in both DRM and SM. However, the highest payoff was the one of the AllC strategy, which prescribes to play C independently from the previous history. Note that, in both DRM and SM, the average cooperation rate in the population of Player 2 was higher than 25%, which implies that full cooperation was indeed the best reply for Player 1.

Table 1. Player 1's strategies: frequency and performance

	Mixture					Outcome			
	Frequency		Payoff Player 1			Frequency		Payoff Player 1	
Strategy	DRM	SM	DRM	SM	Strategy	DRM	SM	DRM	SM
1	0	0	-	-	AllD	0	0	-	-
0.75	0.07	0.38	1.55	2.80	AllC	0.19	0.19	3.25	2.96
0.5	0.45	0.41	2.41	2.84	TfT	0.44	0.81	2.5	2.83
0.25	0.28	0.17	2.89	2.94	Forg	0.15	0	2.48	-
0	0	0	-	-					
other	0.20	0.04	2.35	1.6	other	0.22	0	2.08	-
N	29	29			N	27	27		

2.2 Player 2 strategies

In the Direct Response Method, we classify Players 2 according to their cooperation rate in periods 1-20. If a subject always played C, we classify him as Cooperative. If he cooperated less than 0.25% of the times, we classify him as Non-Cooperative. In all other cases, we classify him as either Mild, Medium, or Hard ExS, depending on whether he cooperated at least 75%, 50% or 25% of the times, respectively. With this classification, Table 2 shows the frequency and the performance of each strategy of Player 2. We note the following:

i) Only Mild ExS in the Mixture treatments were able to gain, on average, more than 4. Specifically, in SM all Mild ExS gained more than 4, with a maximum of 4.5. In DRM, 6 out of the 11 Mild ExS were able to gain more than 4, with a maximum of 4.4.

ii) Mild ExS gain more in the Mixture than in the Outcome treatment (Two-sample Wilcoxon rank-sum (Mann-Whitney) test for the payoff of Mild Ext: DRM p-value= 0.0027, SM p-value= 0.0005).

iii) Hard ExS gain more in the Outcome than in the Mixture treatment (Two-sample Wilcoxon rank-sum (Mann-Whitney) test for Hard ExT: DRM p-value= 0.0500; SM p-value= 0.0679).

iv) Medium ExS gain more in the Mixture in SM, but they gain more in the Outcome in DRM (although both differences are not significant - Two-sample Wilcoxon rank-sum (Mann-Whitney) test: SM p-value= 0.4448; DRM p-value= 0.7131). The result in DRM can be explained by analyzing the behavior of Medium ExS in the Mixture treatment throughout the game. Indeed, among those subjects choosing a Medium ExS, 50% didn't maintain a reputation that was good enough to induce cooperation: at some point during the game, they went below the 25% threshold, and they got an average payoff of 2.74. On the contrary, Medium ExS who never went below the 50% threshold (40%) all managed to get more than 4, resulting in an average payoff of 4.13.

v) According to our model, ExS should be able to gain more than Cooperators in the Mixture, but not in the Outcome treatment. This is true when we consider Mild ExS (Two-sample Wilcoxon rank-sum (Mann-Whitney) test for the difference of payoff between Mild and cooperators, DRM p-values = 0.0388 (Mix), 0.7428 (Out), SM p-values = 0.0001 (Mix), 0.0000 (Out)), but not when we consider Medium or Hard ExS (Two-sample Wilcoxon rank-sum (Mann-Whitney) test for the difference of payoff between Medium and cooperators, DRM p-values= 0.5699 (Mix), 0.8045 (Out), SM p-values = 1.000 (Mix), 0.0000 (Out)).

Table 2. Player 2's strategies: frequency and performance

	Mixture					Outcome			
	Frequency		Payoff Player 2			Frequency		Payoff Player 2	
Strategy	DRM	SM	DRM	SM	Strategy	DRM	SM	DRM	SM
Cooperative	0.14	0.24	3.81	4	Cooperative	0.26	0.48	3.59	4
Mild ExS	0.38	0.52	**4.06**	**4.31**	Mild ExS	0.26	0.19	3.64	3.77
Medium ExS	0.34	0.14	3.27	3.76	Medium ExS	0.19	0.19	3.58	3.39
Hard ExS	0.14	0.07	2.80	1.45	Hard ExS	0.19	0.11	3.57	2.83
Non-Cooperative	0	0.03	-	1	Non-Cooperative	0.11	0.04	3.60	1.63
N	29	29			N	27	27		

3 Supplementary Figures

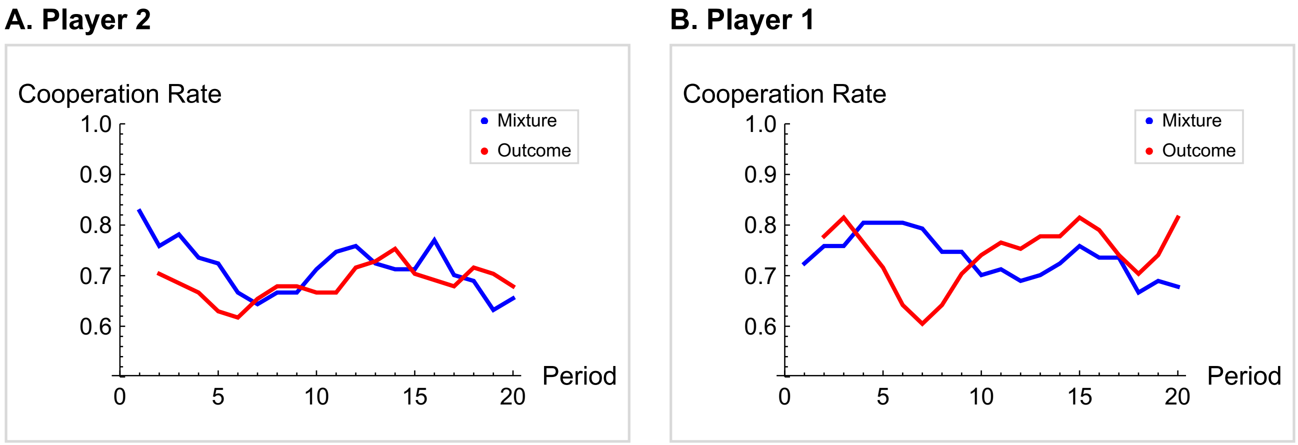

Figure 2. **Cooperation rates over time.** Direct Response Method: players' average cooperation rates, by period, when Player 1 can observe the previous frequency (Mix) or the previous outcome (Out). n(Mixture)=29 for each period; n(Outcome)=27 for each period.

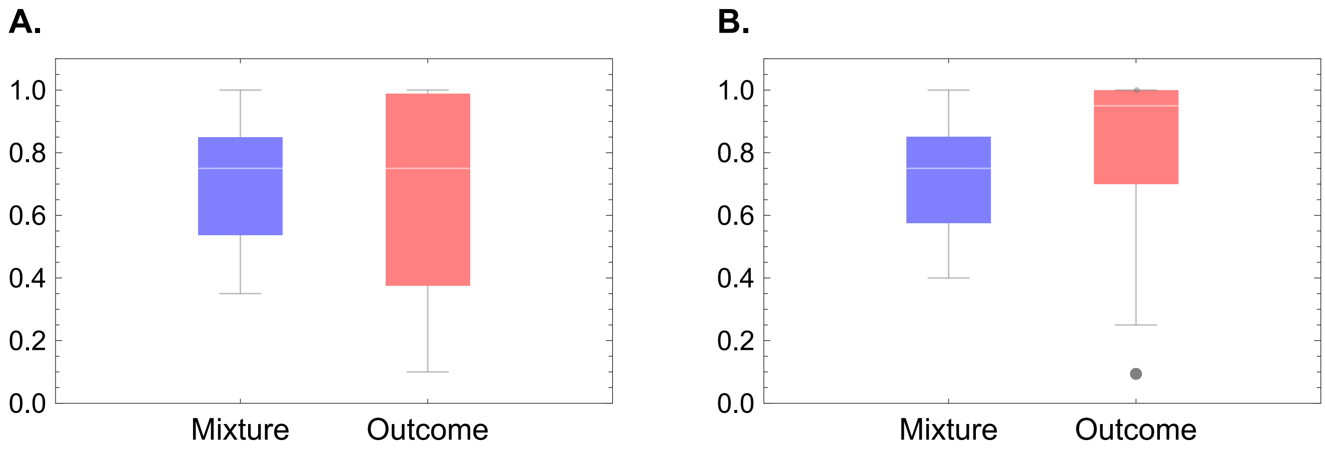

Figure 3. Player 2's strategies. Direct Response Method: distribution of the frequency with which Players 2 played C in the firsts 20 periods of the game, in the Mixture and in the Outcome treatments, when considering all Players 2 (A), or only those who played C at the beginning of the game (B -this could be taken as a proof they they were interested in building a reputation for playing cooperatively). We expect this frequency to be close to 1 when Player 1 can observe the previous action (Outcome treatment), and smaller than 1 when she can observe the previous frequency (Mixture treatment). This is not the case when considering all Players 2 (panel A), as the median choice in the two treatments is the same (.75). However, when we consider only the subset of Players 2 who played C in the first period of the game (panel B), the median in the Outcome treatment (.95) is higher than the median in the Mixture treatment (.75), and the distribution in the former is less dispersed with respect to the case in which all subjects were included in the analysis. Thus, Players 2 who signal in the first period their willingness to build a good reputation are more likely to exploit Players 1 (i.e. they play C with a lower frequency) in the Mixture treatment, that is when extortionate equilibria are possible. Panel A: n(Mixture)=29; n(Outcome)=27. Panel B: n(Mixture)=24; n(Outcome)=19.

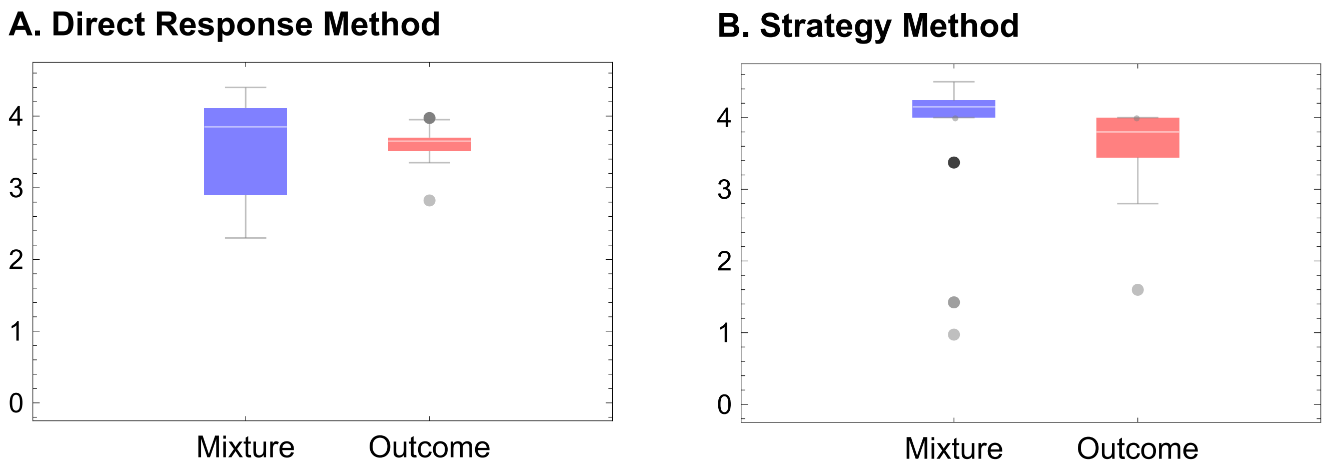

Figure 4. Player 2's payoffs. Distribution of Players 2's payoffs in the two treatments, considering the Direct Response Method (A) and the Strategy Method (B). Two-sample Wilcoxon rank-sum (Mann-Whitney) test: Direct Response Method, $p = 0.0801$; Strategy Method, $p = 0.0002$). n(Mixture)=29; n(Outcome)=27.

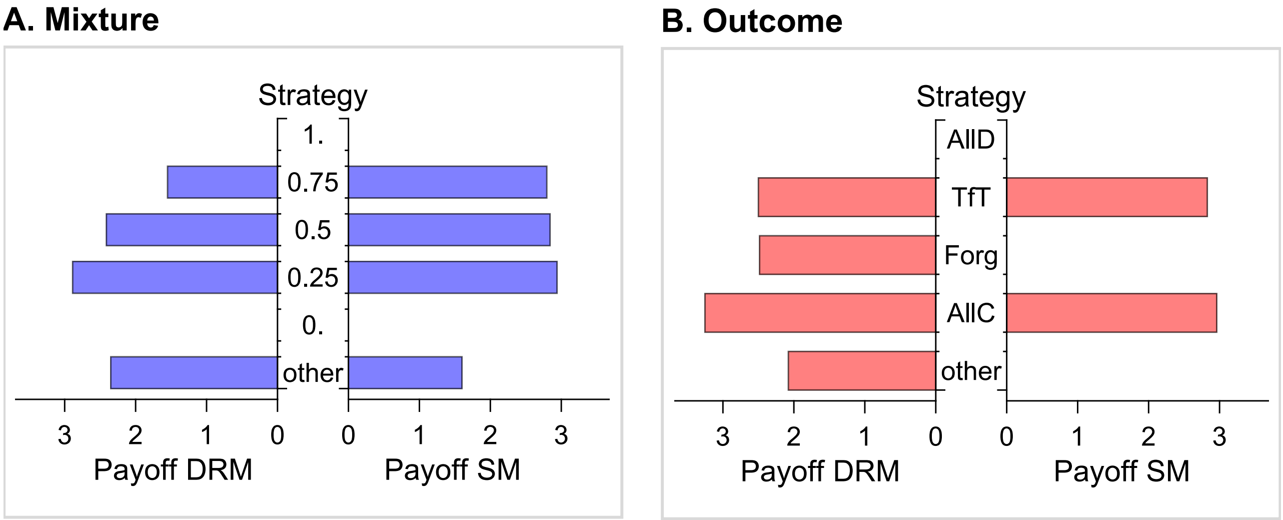

Figure 5. **Player 1's payoffs**. Payoffs of Player 1 in the the two treatments, considering the Direct Response Method (DRM) and the Strategy Method (SM). In the Mixture treatment, the highest payoff was the one of the more tolerant players (that is, those who used a step strategy with a threshold equal to 0.25), while in the Outcome treatment, the highest payoff was the one of the fully cooperative players. n(Mixture)=29; n(Outcome)=27.

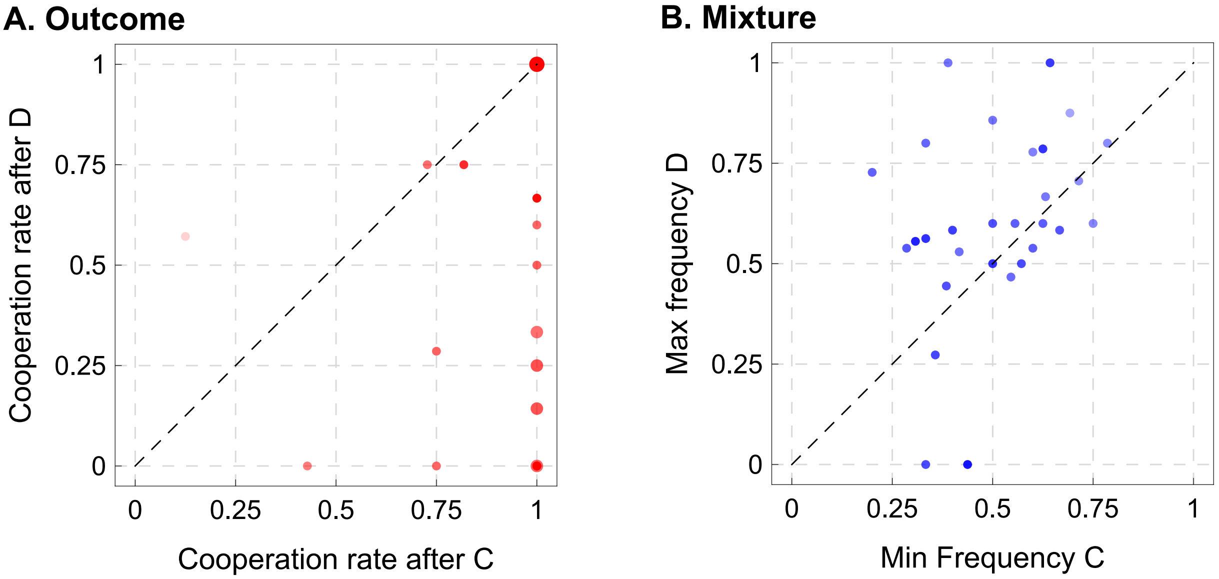

Figure 6. **Player 1's strategies.** Direct Response Method: strategies employed by Player 1, when she can observe the previous outcome (A) or the previous frequency (B). A strategy in the Outcome treatment is represented by the probability to play C after C (x-axes) and after D (y-axes), over periods 6-20. As an example, an unconditional cooperator would be in the top-right corner of the square, while a TfT would be in the bottom-right one. A strategy in the Mixture treatment is represented by the maximum frequency of Player 2's cooperation after which Player 1 chooses D, and by the minimum frequency after which she chooses C. A player using a step function would then be on the diagonal of the square. Each dot is a player; dot's size is proportional to the number of players choosing the same combination, and dot's color is proportional to the average payoff they obtained (with darker colors indicating higher payoffs). The Figure shows that in the Outcome treatment the most used strategy can be considered a mild forgiving TfT: Player 1 always plays C after C, while after D she plays C with a smaller (but often positive) probability - the dots that lie on the y-axes. In the Mixture treatment most of the dots are close to the diagonal, but we find some dots above it, meaning that subjects were punishing Player 2 even when they observed high frequencies of C. n(Mixture)=29; n(Outcome)=27.

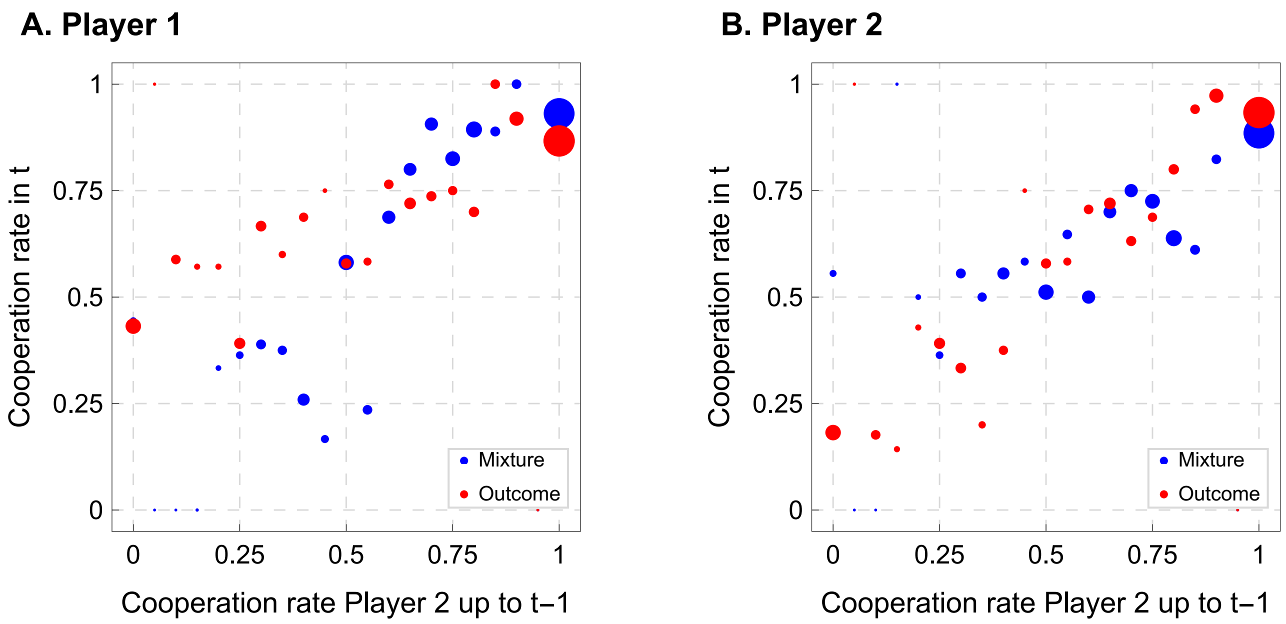

Figure 7. Cooperation rates. Direct Response Method: average cooperation rates in round t (y-axes) of Players 1 (A) and Players 2 (B), given the frequency with which the Player 2 chose C in the previous $t-1$ rounds (q_{t-1}, x-axes). Red dots are from the Outcome treatment (Out), blue dots from the Mixture (Mix). Dot's size is proportional to the number of observations. According to our model, in panel A we should observe a cut off value of q_{t-1} ($\hat{q}$) such that, for values $q_{t-1} < \hat{q}$, the probability that Player 1 plays C should be higher in the Outcome than in the Mixture treatment, while the opposite should hold for $q_{t-1} > \hat{q}$. Moreover, if Player 2 uses an unconditional strategy, we should observe all points in panel B to lie on the diagonal. As we can see, in the Mixture treatment, this is often not the case for low values of q. For example, when the cooperation rate of Player 2 up to period t is 30%, then the probability that Player 2 cooperates in the Outcome treatment is 30%, while the probability that he cooperates in the Mixture treatment is 56% . Since in the Mixture treatment Player 1 can observe the previous frequency, we find that the probability that she cooperates after observing 30% is 33%, while the probability that she cooperates in the Outcome treatment (when she cannot observe the frequency) is 66%.n(Mixture)=551; n(Outcome)=513.

4 Supplementary Tables

Logistic regressions and probability predictions Statistical results for Figure 5. Figure 5 reports the probability of observing the variables on the vertical axes of panels A-C assuming value 1. These probabilities were computed starting from the results of the logit estimations presented in tables 3, 4, 5, where at each period t the independent variable *Cooperation rate* is defined as the fraction of Player 2's cooperative choices from period 1 up to $t-1$. In some cases the best fit is obtained by estimating a quadratic model. Results are robust to clusterization of standard errors at the session level.

Table 3. Cooperation player1

Dep. variable: Cooperation by Player 1	(1) Outcome	(2) Mixture
Cooperation rate	2.190***	-0.180
	(0.300)	(1.988)
Cooperation rate^2		4.298***
		(1.656)
Constant	-0.322	-0.982 *
	(0.201)	(0.575)
N	513	551
LR Chi^2	57.22	144.34
Pseudo R^2	0.096	0.227
$Log-likelihood$	-267.04	-245.39

Standard errors in parentheses

* $p < 0.10$, ** $p < 0.05$, *** $p < 0.01$

The dependent variable is equal to 1 if Player 1 plays C at period t, and zero otherwise.

Table 4. Cooperation Player 2

Dep. variable: Cooperation by Player 2	(1) Outcome	(2) Mixture
Cooperation rate	1.488	-2.329
	(1.345)	(1.717)
Cooperation rate^2	2.639 **	3.836***
	(1.244)	(1.348)
Constant	-1.362***	0.367
	(.314)	(.515)
N	513	551
LR Chi^2	193.26	51.90
Pseudo R^2	0.3020	0.0772
$Log-likelihood$	-223.31	-310.38

Standard errors in parentheses

* $p < 0.10$, ** $p < 0.05$, *** $p < 0.01$

The dependent variable is equal to 1 if Player 2 plays C at period t, and zero otherwise.

Table 5. Outcome CD

Dep. variable: Outcome = CD	(1) Outcome	(2) Mixture
Cooperation rate	2.141*	7.961***
	(1.292)	(2.887)
Cooperation rate^2	-4.612***	-6.328***
	(1.26)	(2.089)
Constant	-0.630**	-3.553***
	(0.274)	(0.939)
N	513	551
LR Chi^2	73.60	12.58
Pseudo R^2	0.148	0.023
$Log-likelihood$	-211.95	-260.81

Standard errors in parentheses

* $p < 0.10$, ** $p < 0.05$, *** $p < 0.01$

The dependent variable is equal to one if the outcome at period t is CD, and zero otherwise.

5 Instructions for the Experiment

Translation of the instructions we used in out experiment. Mixture treatment, Direct Response Method.

Introduction Good Afternoon and thank you for participating in this experiment. It won't be difficult, there won't be any tricky questions, but you have to follow the instructions carefully. Your answers will be anonymous, and it will not be possible to know the identity of who gave the single answer. During the experiment you are not allowed to talk with other participants, and if something is not clear in the instructions, just raise your hand and ask the experimenter for further explanations. If something is not clear, again just raise your hand and ask for explanations to the experimenter.

Participants and roles At the beginning of the experiment half of participants will take the role of "Partecipante UNO" (participant one), and half the role of "Partecipante DUE" (participant two). Each participant will keep his role until the end of the experiment. During the experiment, each Partecipante UNO will be matched with a Partecipante DUE, and both of them will have to make some choices that will allow them to gain some tokens.

Structure of the experiment The experiment consists in two parts, A and B. You will receive more detailed instructions at the beginning of each part. The number of tokens that you will get at the end of the experiment will be determined by the choices that you and the participants with whom you are matched will do. In the two parts of the experiment, you will face the same problem, but you have to make different choices. Specifically:
-in the first part you can make a new choice every time you meet a new participant;
-in the second part you can choose only once, and the same choice will be automatically implemented every time you will meet a new participant

Payments You will receive 3 euro for participating in the experiment. Moreover, during the part A of the experiment you will have the chance to receive some tokens, that will be later conerted in Euro, at the ratio: 1 token=$0,10$ euro. During the part B of the experiment, you will have the chance to receive 10 euro, according to the procedure that we will explain later.

Instructions for the part A of the experiment

Participants and choices The part A of the experiment consists in an indefinite number of rounds. At the beginning of each round, each Partecipante UNO will be matched with a Partecipante DUE. Both participants have to make some choices that will determine the number of tokens they will receive in each round. Specifically:
-the Partecipante UNO has to choose between Dentro (IN) and Fuori (OUT);
-the Partecipante DUE has to choose between Alto (HIGH) and Basso (LOW).

To understand the relation between choices and tokens, during the experiment we will give you a more intuitive representation of payments: a table in which your own payment will always be the first value of each box (in red).

Partecipante UNO \ Partecipante DUE	ALTO	BASSO
DENTRO	4 , 4	0 , 6
FUORI	1 , 1	1 , 1

Partecipante DUE \ Partecipante UNO	DENTRO	FUORI
ALTO	4 , 4	1 ,1
BASSO	6 , 0	1 , 1

The table on the left will be shown to Partecipante UNO, while the one on the right will be shown to Partecipante DUE. In each box there are the number of tokens for Partecipante UNO (first value, in red, in the left table), and the number of tokens for Partecipante DUE (second value, in black, in the left table). Thus:

If UNO chooses Dentro and DUE chooses Alto, both participants get 4 tokens;
If UNO chooses Fuori and DUE chooses Alto, both participants get 1 token;
If UNO chooses Dentro and DUE chooses Basso, UNO gets 0 tokens, and DUE gets 6 tokens;
If UNO chooses Fuori and DUE chooses Basso, both participants get 1 token.

To make your choice you only have to press the button showing your preferred option.

Matching When all participants have made their choices, you will know how many tokens you earned for that round. Then, a new round will start and each Partecipante DUE will be matched with a new Partecipante UNO. Is it possible to meet the same participant again, but you have to wait at least for 10 rounds. Thus, if your role is DUE, and in the present round you meet a particular UNO, you are sure not to meet the same Participante UNO again for at least 10 rounds (the same reasoning applies if your role is UNO).

During the experiment it is not possible to identify a single participant, but every reference will be done to the roles e.g. “you are matched with a new Partecipante UNO (or DUE)”.

How long is the experiment? The experiment consists in an indefinite number of rounds.

You will participate to 20 rounds for sure, after which the experiment will continue with a probability of 50%. The end of the experiment will be thus determined by the result of a random extraction at the end of each round after the 19th. One of the experimenter will put in a box two pieces of papers, with numbers 1 and 2. At the end of each round one number is chosen at random. If the number is 2, the experiment is over. Otherwise, the number is put back in the box and the experiment goes on.

Information At the beginning of each round the Partecipante UNO will receive some information on the past choices of his current opponent. Specifically, the Partecipante UNO will be informed over the percentages with which the Partecipante DUE chose the options Alto and Basso in previous rounds (of course this information will be available only from the second round). For example, the percentages 50%, 50%, imply that the current Partecipante DUE chose Alto and Basso the same number of times in the past. The percentages 33%, 66% imply instead that the current Partecipante DUE chose, on average, Alto twice every three times, and Basso once every three times. To better understand this information, on the monitor of Partecipante UNO will appear a bar graph, indicating the percentages with which the Partecipante DUE chose Alto and Basso in the past:

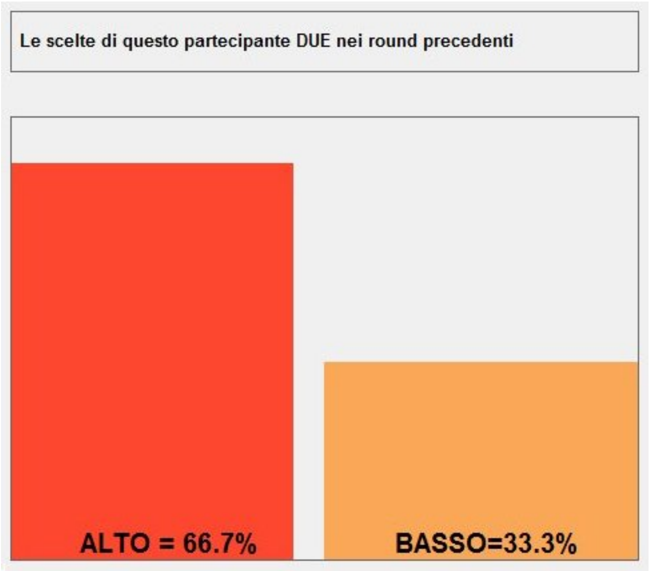

In this example, the bar graph shows the percentages (66.7%, 33.3%), thus the Partecipante DUE chose, on average, Alto twice over three times, and Basso once over three times.

The Partecipante DUE doesn’t get any information over the past behavior of the Partecipante UNO with whom he is matched. On his monitor will appear a synthesis of his own previous choices, represented by the percentages with which he chose Alto and Basso in the previous rounds. Finally, every participant can see a bar showing the average number of tokens earned from the beginning of the experiment until the current round.

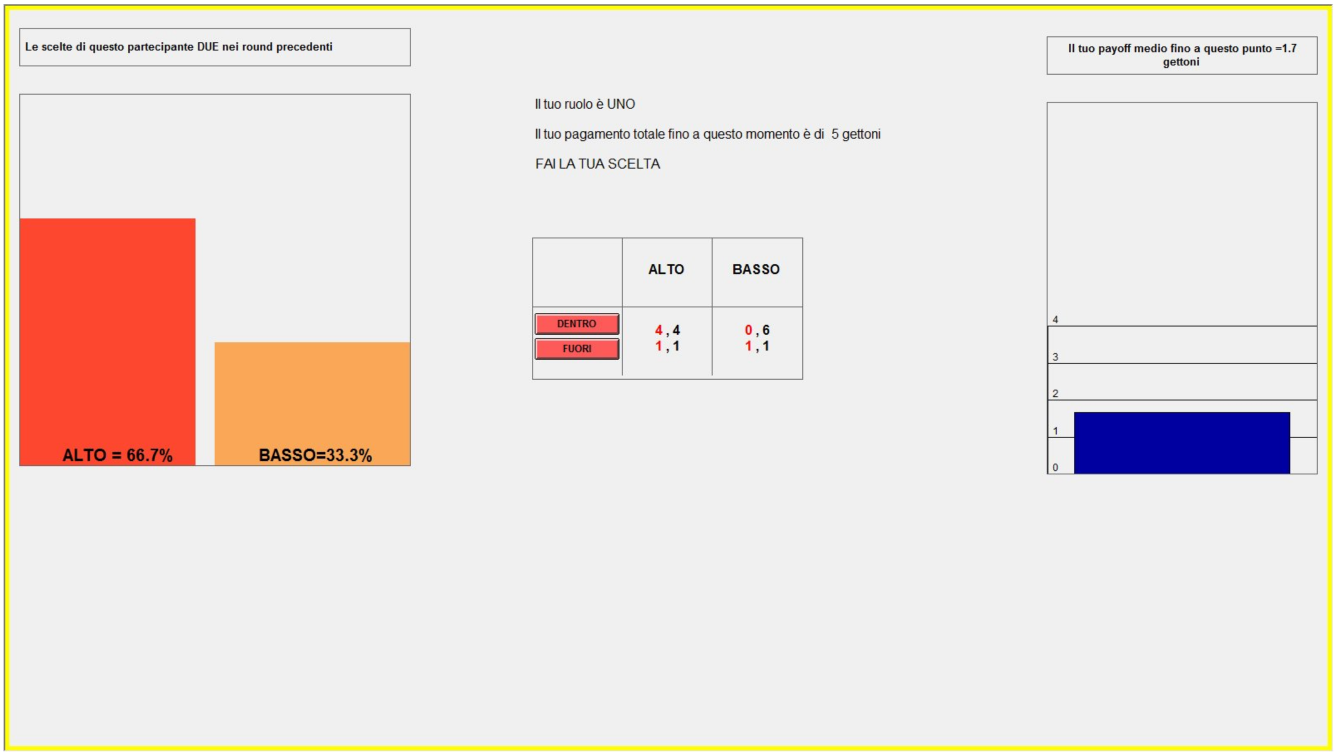

Figure 9. Example of Participante Uno's choice window

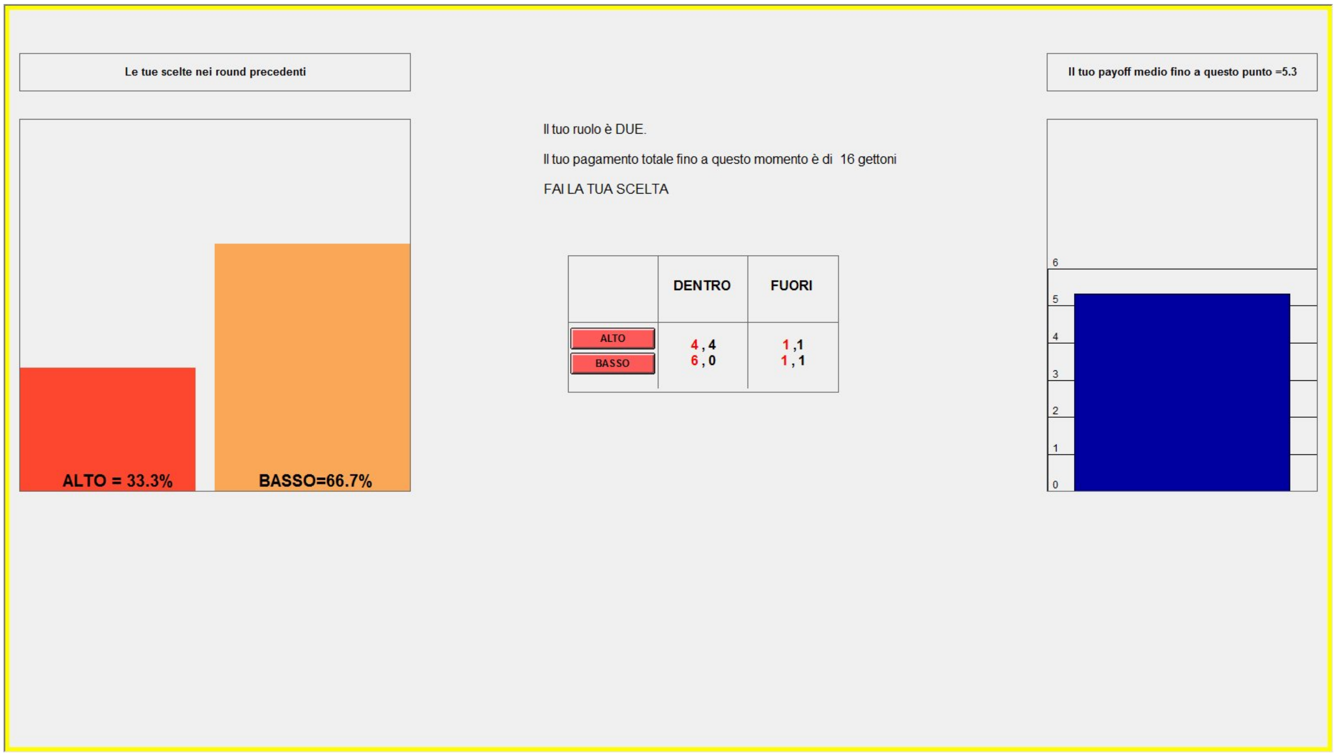

Figure 10. Example of Participante Due's choice window

Payments The total payment is equal to the sum of tokens earned in each round. At the end of the experiment, the total of tokens will be converted in Euros and you will get the corresponding amount of money.

Control Questions

Now we ask you to answer to some control questions.
When all participants will have correctly answered to all questions, the experiment will begin.

1. In one round Partecipante UNO chooses Dentro and Partecipante DUE chooses Alto:
   - How many tokens does Partecipante UNO get in this round?
   - How many tokens does Partecipante DUE get in this round?
2. In one round Partecipante UNO chooses Fuori and Partecipante DUE chooses Alto:
   - How many tokens does Partecipante UNO get in this round?
   - How many tokens does Partecipante DUE get in this round?
3. In every round you will be matched with the same participant. True or False ?
4. At the beginning of every round, Partecipante DUE is informed over Partecipante UNO's previous choices. True or False ?
5. At the beginning of every round, Partecipante UNO is informed over Partecipante DUE's previous choices. True or False ?
6. You are at the first round, which is the probability the experiment will go on?
   • 100% • 30% • 50% • 70% • 0%
7. You are at the 28th round, which is the probability the experiment will go on?
   • 100% • 30% • 50% • 70% • 0%

References

1. Hilbe, C., Traulsen, A. & Sigmund, K. Partners or rivals? strategies for the iterated prisoner's dilemma. *Games Econ. Behav.* **92**, 41–52, DOI: 10.1016/j.geb.2015.05.005 (2015).

2. D'Arcangelo, C. Nash equilibria in memory one strategies for the repeated prisoner's dilemma (2020). Working paper.

3. Dutta, P. K. & Siconolfi, P. Mixed strategy equilibria in repeated games with one-period memory. *Int. J. Econ. Theory* **6**, 167–187 (2010).

4. Ely, J. C. & Valimaki, J. A robust folk theorem for the prisoner's dilemma. *J. Econ. Theory* **102**, 84–105 (2002).
